# Vitamin K intake levels are associated with bone health in people aged over 50 years: a NHANES-based survey

**DOI:** 10.3389/fmed.2024.1485095

**Published:** 2024-11-25

**Authors:** Jiankui Guo, Ziqi Zhou, Jie Gong, Wen Hu, Yuan Liu

**Affiliations:** ^1^Department of Clinical Nutrition, West China Hospital, Sichuan University, Chengdu, China; ^2^Department of Clinical Nutrition, Shenzhen Third People’s Hospital, Shenzhen, China

**Keywords:** vitamin K, osteoporosis, bone loss, bone health, National Health and Nutrition Examination Survey, NHANES

## Abstract

**Background:**

Bone health is important for older adults, and vitamin K (VK) is central to regulating bone formation and promoting bone health. However, whether VK can reduce the risk of osteoporosis and bone loss is unclear. This study hypothesized that different levels of VK intake exert varying effects on bone health in people aged over 50 years.

**Methods:**

Individuals aged above 50 years were recruited from the National Health and Nutrition Examination Survey. VK intake, based on 24-h dietary recall, was divided into three groups, namely the high, medium, and low groups, by sex and tertile. Weighted multiple logistic regression was used to investigate the effects of VK intake on the risk of osteoporosis and bone loss at the femoral neck, trochanter, intertrochanter, total femur, lumbar spine, and overall.

**Results:**

This study included 5,075 individuals. Of them, 1,001 (18%) had osteoporosis (808 women, 83%) and 2,226 (46%) had osteopenia (1,076 women, 54%). Overall, a medium level of VK intake was associated with a reduced risk of bone loss. In women, medium- [odds ratio, OR (95% confidence interval, CI): 0.66(0.47, 0.93)] and high-level [OR (95% CI): 0.71(0.52, 0.98)] VK intake were associated with a decreased risk of osteoporosis. In contrast, only medium-level VK intake was associated with a reduced risk of bone loss [OR (95% CI): 0.58(0.41, 0.81)]. Similar results were obtained for the trochanter, intertrochanter, total femur, and lumbar spine. In men, only medium-level VK intake was associated with a reduced risk of bone loss at the femoral neck [OR (95% CI): 0.66(0.48, 0.90)], whereas high-level VK intake corresponded to a reduced risk of bone loss to the lumbar spine [OR (95% CI): 0.68(0.47, 0.99)]. Nonetheless, VK intake levels did not affect the risk of osteoporosis.

**Conclusion:**

This study demonstrates sex- and bone-site-specific variations in the associations between VK intake levels and bone health in individuals aged over 50 years. Further large-scale cohort studies or randomized controlled trials are warranted to explore the effects of different VK intake levels on bone health in people regardless of their sex and bone site.

## 1 Introduction

Osteoporosis is a common multifactorial systemic metabolic skeletal disorder. It is characterized by low bone mass, degraded skeletal tissue microstructure, bone fragility, and susceptibility to fracture (1–3). As an age-increasing disease, osteoporosis is one of the key causes of disability and mortality in older adults. Its incidence is gradually increasing in older adults, imposing an enormous economic and health burden (4–7). In Europe, osteoporosis affects approximately 32 million people aged over 50 years, resulting in an average of one fracture every 3 s (8, 9). In the US, 10.3% of people aged over 50 years have osteoporosis and 43.9% have osteopenia, with higher rates in women. One in two women and one in four men experience an osteoporosis-related fracture (10–12). Each year, approximately 700,000 osteoporotic vertebral compression fractures are reported in the US alone, imposing economic burdens of approximately $13.8 billion, which will increase with an aging population (13–15).

Osteoporosis is the culmination of multiple factors, such as genetics, individual lifestyle, and nutrition (5). Of them, diet is an important factor affecting bone health. Vitamin K (VK) is a series of derivatives of 2-methyl-1,4-naphthoquinone, which is essential for maintaining normal blood clotting and inhibiting vascular calcification in the body. Moreover, it is central to bone metabolism (16).

Bone metabolism is a repetitive process of bone resorption and formation, termed “bone remodeling.” The appearance and apoptosis of osteoblasts and osteoclasts are essential for maintaining bone remodeling and ensuring bone strength (17). With age, osteoblast production and the bioavailability of testosterone and estradiol decrease, causing the apoptosis of osteoblasts and osteocytes. This results in an imbalance in bone remodeling and progressive bone loss, eventually leading to reduced bone mass and osteoporosis (18). After menopause in women, estrogen levels decrease, which stimulates osteoclast differentiation and activity and inhibits osteoblasts, thereby accelerating bone loss (17, 19). However, VK stimulates osteoblasts and inhibits osteoclasts, thereby promoting bone calcification, which is beneficial for delaying bone loss and reducing the risk of osteoporotic fractures (20). Osteoblasts synthesize VK-dependent gamma-carboxyglutamic acid (Gla) protein, bone Gla protein (BGP or osteocalcin), a low-molecular-weight protein in the region of rapid bone growth. It induces osteoblastic progenitors and regulates calcium phosphate ossification, which serves as a marker of bone formation (21, 22). As a cofactor, VK is required for the *γ*-carboxylation of osteocalcin. Moreover, the glutamate in osteocalcin undergoes γ-carboxylation for osteocalcin to bind calcium ions and hydroxyapatite to mineralize bone and promote bone formation (22–24).

However, the impact of VK on bone health is controversial. VK supplementation maintains bone mineral density (BMD) in postmenopausal women; however, it has also been found that VK supplementation reduces BGP undercarboxylation without affecting BMD (25–29). Additionally, VK supplementation is associated with BGP carboxylation in a dose–response manner; the higher the VK supplementation, the lower the BGP uncarboxylation (30, 31). However, the associations between VK intake levels and the risk of osteoporosis and bone loss are unclear. Therefore, in this cross-sectional study, we hypothesized that different VK intake levels will exert varying effects on osteoporosis or bone loss in individuals aged over 50 years, where higher VK intake promotes or maintains bone health and contributes to a reduced risk of osteoporosis and bone loss.

## 2 Materials and methods

### 2.1 Study participants

The National Health and Nutrition Examination Survey (NHANES) is a complex, multistage, cross-sectional study based on probability sampling. It is updated every 2 years to assess the health and nutritional status of the US population. The NHANES conducts household interviews and physical examinations through the mobile examination center (MEC) throughout the US to collect information on sociodemographics, lifestyle, dietary intake, behavioral status, and medical conditions. The NHANES was approved by the National Center for Health Statistics Ethics Review Board, and all participants signed informed consent forms.

**Figure 1:**
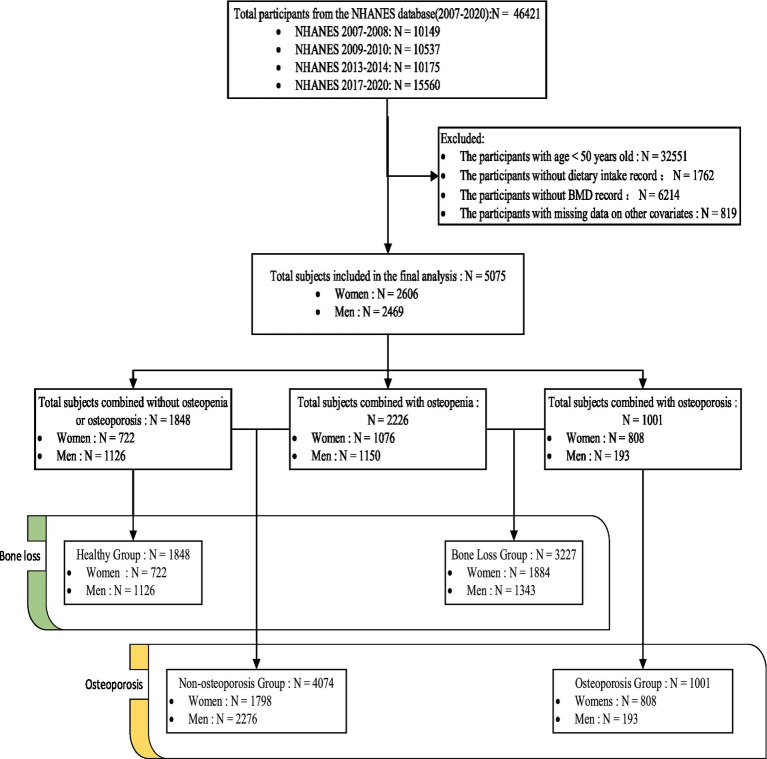
Flow chart of sample screening.

For this study, data from four NHANES cycles (2007–2008, 2009–2010, 2013–2014, and 2017–2020 Prepandemic) were extracted and analyzed (BMD data were unavailable for the 2011–2012 and 2015–2016 cycles). People aged over 50 years with complete dietary and BMD data were included. The exclusion criteria were as follows: missing individuals or who refused to respond to questions about age, sex, income level, education, hypertension, diabetes, smoking status, alcohol consumption, and so on. A total of 46,421 individuals were enrolled in the four cycles. After the screening, 5,075 individuals were finally included. Figure 1 illustrates the flow chart of sample screening. This study used publicly available deidentified data from NHANES; therefore, ethical approval was not needed.

### 2.2 Dietary intake

The NHANES uses the 24-h dietary recall method to investigate the dietary and supplement intake of all participants. The participants received their first 24-h dietary survey at the MEC and completed a second survey via telephonic follow-up within 3 to 10 days. The responses were processed through the US Department of Agriculture’s Food and Nutrition Database for Dietary Studies to calculate the nutrient intake.

Daily intake included dietary and nutritional supplement intake. The average intake over 2 days was calculated as the intake of the participant who responded to 2 days of the dietary survey; the intake over day 1 was calculated as the intake otherwise. Additionally, VK intake was defined as the major exposure factor; vitamin D (VD), vitamin C (VC), calcium, phosphorus, sodium, energy, protein, and caffeine were considered potential confounders for further analysis. Moreover, considering the differences in recommended VK and dietary intake between men and women, VK intake was divided into three levels according to sex and tertile as follows: low (<65.95 mcg/d for women, <69.85 mcg/d for men), medium (≥65.95 mcg/d and < 129.40 mcg/d for women, ≥69.85 mcg/d and < 128.75 mcg/d for men), and high (≥129.40 mcg/d for women, ≥128.75 mcg/d for men) (32).

### 2.3 Defining osteoporosis/osteopenia

Dual-energy X-ray absorptiometry (DXA) was utilized in the NHANES to measure BMD. The examination was conducted via a Hologic QDR-4500A sector beam densitometer (Hologic; Bedford, MA, USA), and all data were analyzed via Hologic APEX software (version 4.0).

The participants were diagnosed as healthy or with osteopenia/osteoporosis. All participants were defined as having osteoporosis/osteopenia based on osteoporosis (OSQ) questionnaires and the BMD of the femoral neck, trochanter, intertrochanter, total femur, and lumbar spine. The lumbar spine BMD was determined by calculating the mean BMD from L1 to L4. The participants were diagnosed with osteoporosis/osteopenia if they met the diagnostic criteria for osteoporosis/osteopenia at any site, and the remaining were considered healthy. Osteoporosis/osteopenia was diagnosed according to the WHO’s recommendation, with the mean BMD of non-Hispanic white people aged 20–29 years in NHANES III used as the reference value (33–35). A BMD ≥2.5 standard deviations (SD) below the reference value indicated osteoporosis, whereas a BMD between 1 and 2.5 SD of the reference value indicated osteopenia (36). Participants with osteoporosis were also defined as those who reported an osteoporosis diagnosis in the OSQ questionnaire. The participants were divided into osteoporosis and non-osteoporosis (including healthy and osteopenia) groups to analyze the associations between VK intake and the risk of osteoporosis; and they were divided into the healthy and bone loss (including osteopenia and osteoporosis) groups to analyze the associations between VK intake and the risk of bone loss.

### 2.4 Covariates

Considering the potential influence of other factors, the following covariates were included: age (50–59, 60–69, 70–79, ≥80 years), race (non-Hispanic White, non-Hispanic Black, Mexican American, other Hispanic, and other race/multiracial), education level (less than college, college or above), income level (based on the ratio of family income to poverty: low (<1.3), medium (≥1.3 and < 3.5), and high (≥3.5)), body mass index (BMI; underweight, normal, overweight, and obesity), smoking status (never smoked, ever smoked, and current smoker), drinking status (never drank, nondrinking past 12 months, ≤1 drink per month, and > 1 drink per month), diabetes status (diabetes, prediabetes, and healthy), hypertension status (yes, no), sedentary time (<8 h, ≥8 h), family history of osteoporosis (yes, no), history of prednisone or cortisone use (yes, no), history of estrogen use (yes, no), and dietary intake of VD (mcg/d), VC (mg/d), calcium (mg/d), phosphorus (mg/d), sodium (mg/d), energy (kcal/d), protein (g/d), and caffeine (mg/d).

In the NHANES, data were collected at all study sites by trained personnel with standardized procedures. Dietary intake data were obtained from the Dietary Interview - Total Nutrient Intakes, First/Second Day (DR1TOT/DR2TOT) and the Dietary Supplement Use 24-Hour - Total Dietary Supplements, First/Second Day (DS1TOT/DS2TOT) documents. They were processed and included in the model as continuous variables for subsequent analysis. Age, race, education, and income level were collected from the Demographic Variables and Sample Weights (DEMO) file; BMI from the Body Measures (BMX) file; smoking status from the Smoking - Cigarette Use (SMQ) file; drinking status from the Alcohol Use (ALQ) file; diabetes status from the Diabetes (DIQ) file; hypertension status from the Blood Pressure and Cholesterol (BPQ) file; sedentary time from the Physical Activity (PAQ) file; family history of osteoporosis and a history of prednisone or cortisone use from the OSQ file; and history of estrogen use from the Reproductive Health (RHQ) file. These data were categorized according to the preceding text and included as categorical variables.

### 2.5 Statistical analysis

For all calculations and analyses, the NHANES sample weights were used. The weights were selected according to the NHANES database instructions (37). They were adjusted for the weight of the first day 24-h dietary recall (WTDRD1) because this study was based on data from the first day of the dietary recall. Four NHANES periods were combined, totaling 9.2 years. Continuous variables are reported as *median (P_25_, P_75_)*, and categorical variables are reported as unweighted numbers and weighted proportions. Sex was used for the subgroup analysis. A restricted cubic spline (RCS) analysis was conducted to explore the association between VK intake and bone health with a node number of 5. Weighted multiple logistic regression was used to explore the associations between different VK intake levels and the overall risk of osteoporosis or bone loss and that at specific sites. Five sets of models were constructed with osteoporosis or bone loss as the dependent variable, the nonosteoporosis or healthy group as the reference, and the VK intake level as the predictor. Model 1 was unadjusted; Model 2 was adjusted for age and race; Model 3 included Model 2 and was additionally adjusted for education, poverty, BMI, sedentary behavior, a history of smoking, and a history of alcohol use; Model 4 included Model 3 and was additionally adjusted for a family history of osteoporosis and a history of cortisone use, diabetes mellitus, and hypertension, and estrogen use in women; Model 5 added dietary variables, including the intake of VD, VC, calcium, phosphorus, sodium, energy, protein, and caffeine, to Model 4.

All statistical analyses were conducted with R software (version 4.2.3, Vienna, Austria). A *p* < 0.05 indicated statistical significance; the Bonferroni correction was used for multiple comparisons. The Strengthening the Reporting of Observational Studies in Epidemiology-Nutritional Epidemiology (STROBE-NUT) guidelines were followed (38).

## 3 Results

### 3.1 Baseline information

A total of 5,075 participants were finally analyzed; of them, 2,606 (55%) were women, 1,001 (18%) had osteoporosis, and 2,226 (46%) had osteopenia. The baseline characteristics, such as age and other confounders, differed between the participants with different bone health (healthy, osteopenia, and osteoporosis) (*p* < 0.05) (Supplementary Table A), in addition to sex differences in bone health, baseline information, and confounders (*p* < 0.05) (Supplementary Table B). The RCS results demonstrated a non-linear association between VK intake levels and bone health (Supplementary Figure 1). The baseline conditions overall and by sex after generalized grouping by bone health (nonosteoporosis vs. osteoporosis, healthy vs. bone loss) are presented follows (Tables 1, 2).

**Table 1:** Baseline characteristics of participants in the osteoporosis and non-osteoporosis groups.

		Bone health groups		
Characteristic	Overall^1^	Nonosteoporosis^1^	Osteoporosis^1^	*P* ^2^
All participants				
N (*n*, %)	5,075 (100%)	4,074 (82%)	1,001 (18%)	
Age (*n*, %)				**<0.001**
50–59 years	2,011 (50%)	1,760 (55%)	251 (31%)	
60–69 years	1,837 (30%)	1,481 (30%)	356 (33%)	
70–79 years	873 (14%)	623 (12%)	250 (24%)	
80+ years	354 (5.2%)	210 (3.6%)	144 (12%)	
Gender (*n*, %)				**<0.001**
Men	2,469 (45%)	2,276 (52%)	193 (17%)	
Women	2,606 (55%)	1,798 (48%)	808 (83%)	
RACE (*n*, %)				**0.008**
Non-Hispanic White	2,316 (73%)	1,807 (73%)	509 (74%)	
Non-Hispanic Black	1,072 (9.7%)	940 (10%)	132 (6.6%)	
Mexican American	705 (5.4%)	564 (5.4%)	141 (5.6%)	
Other Hispanic	524 (4.6%)	420 (4.4%)	104 (5.1%)	
Other race/multiracial	458 (7.0%)	343 (6.5%)	115 (9.2%)	
Education level (*n*, %)				**<0.001**
Less than college	2,428 (40%)	1,892 (38%)	536 (47%)	
College or above	2,647 (60%)	2,182 (62%)	465 (53%)	
Income level (*n*, %)				**<0.001**
Low income	1,337 (16%)	1,022 (15%)	315 (23%)	
Middle income	1,910 (31%)	1,520 (30%)	390 (36%)	
High income	1,828 (52%)	1,532 (55%)	296 (41%)	
Smoke (*n*, %)				**<0.001**
Never smoking	2,658 (54%)	2,083 (53%)	575 (58%)	
Used smoking	1,605 (32%)	1,349 (33%)	256 (24%)	
Now smoking	812 (15%)	642 (14%)	170 (17%)	
Drink (*n*, %)				**<0.001**
Never drinking	677 (11%)	461 (9.3%)	216 (16%)	
Non-drink Past 12 Mon	1,238 (19%)	972 (18%)	266 (27%)	
≤1 Drink/mon	1,301 (26%)	1,033 (26%)	268 (26%)	
>1 Drink/mon	1,859 (44%)	1,608 (47%)	251 (31%)	
BMI (*n*, %)				**<0.001**
Normal	1,312 (27%)	916 (23%)	396 (40%)	
Underweight	82 (1.4%)	43 (0.7%)	39 (4.4%)	
Overweight	1,939 (38%)	1,589 (39%)	350 (36%)	
Obesity	1,742 (34%)	1,526 (37%)	216 (19%)	
Sedentary time (*n*, %)				**0.023**
< 8 h	3,606 (66%)	2,877 (66%)	729 (71%)	
≥8 h	1,469 (34%)	1,197 (34%)	272 (29%)	
Diabetes (*n*, %)				0.704
Healthy	3,988 (83%)	3,186 (83%)	802 (83%)	
Prediabetes	165 (3.0%)	138 (3.1%)	27 (2.4%)	
Diabetes	922 (14%)	750 (14%)	172 (14%)	
Hypertension (*n*, %)				0.463
Healthy	2,555 (56%)	2,069 (56%)	486 (54%)	
Hypertension	2,520 (44%)	2,005 (44%)	515 (46%)	
Family history of osteoporosis (Yes, *n*, %)	706 (18%)	487 (16%)	219 (28%)	**<0.001**
History of prednisone or cortisone use (Yes, *n*, %)	317 (6.4%)	213 (5.4%)	104 (11%)	**<0.001**
History of estrogen use (Yes, *n*, %)	899 (21%)	621 (19%)	278 (31%)	**<0.001**
VD (mcg/d)	9.7 (3.2, 26.0)	8.8 (3.1, 24.5)	13.7 (3.7, 31.6)	**<0.001**
VC (mg/d)	107.4 (46.9, 198.0)	106.5 (48.3, 195.4)	111.3 (41.3, 212.0)	0.812
Calcium (mg/d)	1,027.9 (688.0, 1454.8)	1,028.5 (688.0, 1,434.6)	1,017.2 (723.7, 1562.5)	0.121
Phosphorus (mg/d)	1,253.0 (959.0, 1575.5)	1,290.0 (980.5, 1614.0)	1,114.9 (885.9, 1417.2)	**<0.001**
Sodium (mg/d)	3,011.5 (2300.8, 3929.9)	3,097.2 (2390.6, 4040.0)	2,659.2 (2051.6, 3412.7)	**<0.001**
Energy(kcal/d)	1,863.5 (1,466.3, 2384.1)	1,921.5 (1,496.0, 2446.1)	1,673.8 (1334.5, 2086.4)	**<0.001**
Protein (g/d)	73.1 (56.3, 94.0)	74.8 (57.7, 95.7)	63.9 (50.3, 79.8)	**<0.001**
Caffeine (mg/d)	145.5 (57.5, 257.0)	154.0 (63.0, 267.6)	107.5 (37.0, 228.0)	**<0.001**
VK (mcg/d)	93.8 (57.1, 162.8)	96.1 (59.6, 165.9)	87.2 (51.3, 141.0)	**<0.001**
Level of VK intake (*n*, %)				**<0.001**
Low	2,044 (33%)	1,599 (31%)	445 (40%)	
Medium	1,558 (33%)	1,273 (34%)	285 (29%)	
High	1,473 (34%)	1,202 (35%)	271 (30%)	
Men				
N (*n*, %)	2,469 (100%)	2,276 (93%)	193 (6.9%)	
Age (*n*, %)				**<0.001**
50–59 years	995 (53%)	942 (54%)	53 (39%)	
60–69 years	887 (29%)	826 (29%)	61 (30%)	
70–79 years	426 (13%)	381 (13%)	45 (20%)	
80+ years	161 (4.1%)	127 (3.6%)	34 (11%)	
Race (*n*, %)				0.416
Non-Hispanic White	1,106 (74%)	1,006 (73%)	100 (74%)	
Non-Hispanic Black	544 (9.8%)	514 (10%)	30 (7.4%)	
Mexican American	356 (5.8%)	333 (5.8%)	23 (5.8%)	
Other Hispanic	249 (4.3%)	232 (4.3%)	17 (3.2%)	
Other race/multiracial	214 (6.6%)	191 (6.4%)	23 (9.4%)	
Education level (*n*, %)				0.253
Less than college	1,226 (41%)	1,127 (40%)	99 (46%)	
College or above	1,243 (59%)	1,149 (60%)	94 (54%)	
Income level (*n*, %)				0.160
Low income	641 (16%)	577 (15%)	64 (22%)	
Middle income	909 (29%)	844 (30%)	65 (25%)	
High income	919 (55%)	855 (55%)	64 (53%)	
Smoke (*n*, %)				0.161
Never smoking	1,037 (46%)	964 (46%)	73 (44%)	
Used smoking	975 (38%)	904 (38%)	71 (33%)	
Now smoking	457 (16%)	408 (15%)	49 (22%)	
Drink (*n*, %)				**0.007**
Never drinking	158 (5.7%)	142 (5.4%)	16 (10.0%)	
Non-drink past 12 mon	648 (20%)	587 (19%)	61 (34%)	
≤1 Drink/Mon	533 (20%)	490 (20%)	43 (18%)	
>1 Drink/Mon	1,130 (54%)	1,057 (56%)	73 (38%)	
BMI (*n*, %)				**<0.001**
Normal	611 (23%)	513 (21%)	98 (49%)	
Underweight	34 (0.8%)	28 (0.5%)	6 (5.0%)	
Overweight	1,038 (42%)	982 (43%)	56 (26%)	
Obesity	786 (35%)	753 (36%)	33 (20%)	
Sedentary time (*n*, %)				0.484
< 8 h	1,740 (66%)	1,613 (66%)	127 (62%)	
≥8 h	729 (34%)	663 (34%)	66 (38%)	
Diabetes (*n*, %)				0.115
Healthy	1,885 (80%)	1,742 (80%)	143 (74%)	
Prediabetes	89 (3.1%)	84 (3.2%)	5 (2.2%)	
Diabetes	495 (17%)	450 (16%)	45 (24%)	
Hypertension (*n*, %)				0.821
Healthy	1,288 (55%)	1,186 (55%)	102 (56%)	
Hypertension	1,181 (45%)	1,090 (45%)	91 (44%)	
Family history of osteoporosis (Yes, *n*, %)	238 (11%)	208 (11%)	30 (20%)	**0.013**
History of prednisone or cortisone use (Yes, *n*, %)	116 (5.3%)	97 (4.7%)	19 (12%)	**0.002**
VD (mcg/d)	7.5 (3.1, 18.0)	7.1 (3.1, 17.4)	10.3 (3.0, 30.8)	0.052
VC (mg/d)	106.7 (45.7, 189.9)	104.8 (45.0, 185.6)	129.5 (60.1, 247.0)	0.107
Calcium (mg/d)	1,022.0 (697.2, 1402.8)	1,022.0 (694.0, 1385.9)	1,009.8 (733.4, 1622.5)	0.249
Phosphorus (mg/d)	1,432.0 (1,117.6, 1764.8)	1,433.6 (1,123.5, 1767.4)	1,385.0 (1,003.2, 1699.8)	0.106
Sodium (mg/d)	3,507.0 (2,739.9, 4479.7)	3,519.9 (2,741.4, 4476.6)	3,431.4 (2,688.2, 4493.7)	0.576
Energy(kcal/d)	2,176.6 (1,728.7, 2700.3)	2,185.1 (1,730.5, 2703.6)	2,033.8 (1,658.4, 2691.6)	0.281
Protein (g/d)	84.9 (64.4, 107.9)	85.1 (65.4, 107.7)	76.7 (55.4, 108.7)	0.068
Caffeine (mg/d)	171.5 (68.5, 294.9)	173.0 (70.5, 297.5)	119.8 (29.1, 275.3)	0.051
VK (mcg/d)	95.2 (59.5, 163.7)	95.2 (59.8, 163.4)	92.7 (57.5, 165.8)	0.979
Level of VK intake (*n*, %)				0.621
Low	1,008 (33%)	921 (32%)	87 (36%)	
Medium	760 (33%)	709 (33%)	51 (29%)	
High	701 (34%)	646 (34%)	55 (35%)	
Women				
N (*n*, %)	2,606 (100%)	1798 (72%)	808 (28%)	
Age (*n*, %)				**<0.001**
50-59 years	1,016 (48%)	818 (55%)	198 (29%)	
60-69 years	950 (31%)	655 (30%)	295 (34%)	
70-79 years	447 (15%)	242 (12%)	205 (25%)	
80+ years	193 (6.1%)	83 (3.6%)	110 (12%)	
Race (*n*, %)				**0.012**
NON-Hispanic White	1,210 (73%)	801 (73%)	409 (73%)	
NON-Hispanic Black	528 (9.6%)	426 (11%)	102 (6.4%)	
Mexican American	349 (5.1%)	231 (4.9%)	118 (5.5%)	
Other Hispanic	275 (4.8%)	188 (4.6%)	87 (5.4%)	
Other race/multiracial	244 (7.4%)	152 (6.7%)	92 (9.2%)	
Education level (*n*, %)				**<0.001**
Less than college	1,202 (39%)	765 (36%)	437 (47%)	
College or above	1,404 (61%)	1,033 (64%)	371 (53%)	
Income level (*n*, %)				**<0.001**
Low income	696 (17%)	445 (14%)	251 (23%)	
Middle income	1,001 (33%)	676 (31%)	325 (39%)	
High income	909 (50%)	677 (55%)	232 (38%)	
Smoke (*n*, %)				0.130
Never smoking	1,621 (60%)	1,119 (59%)	502 (61%)	
Used smoking	630 (26%)	445 (28%)	185 (23%)	
Now smoking	355 (14%)	234 (13%)	121 (16%)	
Drink (*n*, %)				**<0.001**
Never drinking	519 (15%)	319 (14%)	200 (17%)	
Non-drink past 12 mon	590 (19%)	385 (16%)	205 (26%)	
≤1 Drink/Mon	768 (31%)	543 (32%)	225 (28%)	
>1 Drink/Mon	729 (36%)	551 (39%)	178 (29%)	
BMI (*n*, %)				**<0.001**
Normal	701 (30%)	403 (27%)	298 (38%)	
Underweight	48 (1.9%)	15 (0.9%)	33 (4.3%)	
Overweight	901 (35%)	607 (34%)	294 (39%)	
Obesity	956 (33%)	773 (39%)	183 (19%)	
Sedentary time (*n*, %)				**0.027**
< 8 h	1,866 (67%)	1,264 (65%)	602 (72%)	
≥8 h	740 (33%)	534 (35%)	206 (28%)	
Diabetes (*n*, %)				0.728
Healthy	2,103 (86%)	1,444 (86%)	659 (85%)	
Prediabetes	76 (2.9%)	54 (3.1%)	22 (2.5%)	
Diabetes	427 (11%)	300 (11%)	127 (12%)	
Hypertension (*n*, %)				0.195
Healthy	1,267 (57%)	883 (58%)	384 (54%)	
Hypertension	1,339 (43%)	915 (42%)	424 (46%)	
Family history of osteoporosis (Yes, *n*, %)	468 (23%)	279 (21%)	189 (29%)	**0.002**
History of prednisone or cortisone use (Yes, *n*, %)	201 (7.3%)	116 (6.0%)	85 (11%)	**<0.001**
History of estrogen use (Yes, *n*, %)	899 (39%)	621 (39%)	278 (38%)	0.568
VD (mcg/d)	12.9 (3.5, 29.7)	12.2 (3.4, 29.2)	14.3 (3.8, 31.7)	0.148
VC (mg/d)	108.1 (48.3, 210.3)	107.5 (51.0, 212.1)	108.3 (40.8, 204.6)	0.199
Calcium (mg/d)	1034.1 (687.0, 1516.1)	1035.3 (680.5, 1,504.0)	1027.0 (719.1, 1,550.7)	0.567
Phosphorus (mg/d)	1125.0 (885.1, 1404.8)	1144.0 (890.1, 1,418.0)	1092.6 (868.5, 1352.6)	0.053
Sodium (mg/d)	2692.4 (2091.3, 3398.6)	2754.7 (2153.3, 3461.6)	2491.7 (1978.5, 3214.9)	**<0.001**
Energy(kcal/d)	1,653.4 (1,346.2, 2061.0)	1693.0 (1351.3, 2086.2)	1617.1 (1307.0, 1994.0)	**0.026**
Protein (g/d)	64.0 (50.9, 81.4)	65.3 (51.2, 82.8)	62.2 (50.1, 76.0)	**0.012**
Caffeine (mg/d)	129.5 (50.0, 234.8)	139.5 (54.7, 240.0)	104.5 (37.5, 218.3)	**0.006**
VK (mcg/d)	92.7 (54.8, 161.0)	96.7 (59.4, 171.1)	83.3 (49.9, 140.7)	**<0.001**
Level of VK intake (*n*, %)				**<0.001**
Low	1,036 (33%)	678 (30%)	358 (41%)	
Medium	798 (33%)	564 (34%)	234 (29%)	
High	772 (34%)	556 (36%)	216 (30%)	

^1^Median (P25, P75) for continuous; *n* (%) for categorical. ^2^Chi-squared test with Rao and Scott’s second-order correction; Wilcoxon rank-sum test for complex survey samples. BMI, body mass index; VD, vitamin D; VC, vitamin C; VK, vitamin K. Bold values indicate *p* < 0.05.

**Table 2:** Baseline characteristics of the participants in the bone loss and healthy groups.

		Bone Health Groups		
Characteristic	Overall^1^	Healthy^1^	Bone loss^1^	*P* ^2^
All participants				
N (*n*, %)	5,075 (100%)	1,848 (36%)	3,227 (64%)	
Age (*n*, %)				**<0.001**
50–59 years	2,011 (50%)	912 (61%)	1,099 (44%)	
60–69 years	1,837 (30%)	652 (28%)	1,185 (32%)	
70–79 years	873 (14%)	224 (9.0%)	649 (17%)	
80+ years	354 (5.2%)	60 (2.0%)	294 (7.0%)	
Gender (*n*, %)				**<0.001**
Men	2,469 (45%)	1,126 (59%)	1,343 (38%)	
Women	2,606 (55%)	722 (41%)	1,884 (62%)	
Race (*n*, %)				**<0.001**
NON-Hispanic White	2,316 (73%)	719 (70%)	1,597 (75%)	
NON-Hispanic Black	1,072 (9.7%)	571 (15%)	501 (6.9%)	
Mexican American	705 (5.4%)	262 (5.6%)	443 (5.3%)	
Other Hispanic	524 (4.6%)	178 (3.7%)	346 (5.0%)	
Other Race/Multiracial	458 (7.0%)	118 (5.7%)	340 (7.8%)	
Education level (*n*, %)				0.458
Less than college	2,428 (40%)	864 (41%)	1,564 (39%)	
College or above	2,647 (60%)	984 (59%)	1,663 (61%)	
Income level (*n*, %)				**0.038**
Low income	1,337 (16%)	450 (15%)	887 (17%)	
Middle income	1,910 (31%)	673 (29%)	1,237 (33%)	
High income	1,828 (52%)	725 (56%)	1,103 (50%)	
Smoke (*n*, %)				0.080
Never smoking	2,658 (54%)	966 (55%)	1,692 (53%)	
Used smoking	1,605 (32%)	598 (33%)	1,007 (31%)	
Now smoking	812 (15%)	284 (12%)	528 (16%)	
Drink (*n*, %)				**<0.001**
Never drinking	677 (11%)	186 (8.6%)	491 (12%)	
Non-drink past 12 mon	1,238 (19%)	435 (17%)	803 (21%)	
≤1 Drink/Mon	1,301 (26%)	466 (25%)	835 (26%)	
>1 Drink/Mon	1,859 (44%)	761 (50%)	1,098 (41%)	
BMI (*n*, %)				**<0.001**
Normal	1,312 (27%)	239 (12%)	1,073 (35%)	
Underweight	82 (1.4%)	5 (0.1%)	77 (2.1%)	
Overweight	1,939 (38%)	685 (38%)	1,254 (38%)	
Obesity	1,742 (34%)	919 (50%)	823 (25%)	
Sedentary time (*n*, %)				0.738
< 8 h	3,606 (66%)	1,304 (67%)	2,302 (66%)	
≥8 h	1,469 (34%)	544 (33%)	925 (34%)	
Diabetes (*n*, %)				**0.008**
Healthy	3,988 (83%)	1,399 (80%)	2,589 (85%)	
Prediabetes	165 (3.0%)	74 (4.0%)	91 (2.5%)	
Diabetes	922 (14%)	375 (16%)	547 (12%)	
Hypertension (*n*, %)				**0.015**
Healthy	2,555 (56%)	878 (52%)	1,677 (58%)	
Hypertension	2,520 (44%)	970 (48%)	1,550 (42%)	
Family history of osteoporosis (Yes, *n*, %)	706 (18%)	202 (12%)	504 (21%)	**<0.001**
History of prednisone or cortisone use (Yes, *n*, %)	317 (6.4%)	85 (4.7%)	232 (7.3%)	**0.009**
History of estrogen use (Yes, *n*, %)	899 (21%)	229 (15%)	670 (25%)	**<0.001**
VD (mcg/d)	9.7 (3.2, 26.0)	8.0 (3.2, 21.8)	10.7 (3.2, 27.4)	0.069
VC (mg/d)	107.4 (46.9, 198.0)	107.8 (46.6, 185.1)	107.0 (47.4, 203.7)	0.620
Calcium (mg/d)	1,027.9 (688.0, 1,454.8)	1,028.5 (693.3, 1,406.3)	1,022.0 (686.2, 1,484.5)	0.680
Phosphorus (mg/d)	1,253.0 (959.0, 1,575.5)	1,346.9 (1,021.5, 1,659.5)	1,199.8 (935.0, 1,523.2)	**<0.001**
Sodium (mg/d)	3,011.5 (2,300.8, 3,929.9)	3,211.0 (2,520.8, 4,200.3)	2,909.4 (2,228.5, 3,756.2)	**<0.001**
Energy(kcal/d)	1,863.5 (1,466.3, 2,384.1)	2,011.0 (1,575.0, 2,506.1)	1,802.0 (1,411.5, 2,291.3)	**<0.001**
Protein (g/d)	73.1 (56.3, 94.0)	78.3 (60.0, 98.3)	69.3 (53.9, 90.2)	**<0.001**
Caffeine (mg/d)	145.5 (57.5, 257.0)	156.0 (57.1, 272.0)	142.0 (57.5, 249.0)	0.151
VK (mcg/d)	93.8 (57.1, 162.8)	96.1 (62.5, 157.2)	92.6 (53.3, 166.0)	0.394
Level of VK intake (*n*, %)				0.084
Low	2,044 (33%)	730 (30%)	1,314 (34%)	
Medium	1,558 (33%)	600 (36%)	958 (31%)	
High	1,473 (34%)	518 (34%)	955 (34%)	
Men				
N (*n*, %)	2,469 (100%)	1,126 (46%)	1,343 (54%)	
Age (*n*, %)				**<0.001**
50–59 years	995 (53%)	529 (62%)	466 (46%)	
60–69 years	887 (29%)	408 (27%)	479 (31%)	
70–79 years	426 (13%)	143 (8.5%)	283 (17%)	
80+ years	161 (4.1%)	46 (2.5%)	115 (5.5%)	
Race (*n*, %)				**<0.001**
NON-Hispanic White	1,106 (74%)	439 (71%)	667 (76%)	
NON-Hispanic Black	544 (9.8%)	326 (13%)	218 (6.7%)	
Mexican American	356 (5.8%)	168 (5.9%)	188 (5.8%)	
Other Hispanic	249 (4.3%)	116 (4.0%)	133 (4.5%)	
Other Race/Multiracial	214 (6.6%)	77 (6.2%)	137 (6.9%)	
Education level (*n*, %)				0.512
Less than college	1,226 (41%)	562 (42%)	664 (40%)	
College or above	1,243 (59%)	564 (58%)	679 (60%)	
Income level (*n*, %)				0.892
Low income	641 (16%)	279 (16%)	362 (16%)	
Middle income	909 (29%)	399 (29%)	510 (30%)	
High income	919 (55%)	448 (56%)	471 (55%)	
Smoke (*n*, %)				**0.016**
Never smoking	1,037 (46%)	526 (51%)	511 (42%)	
Used smoking	975 (38%)	409 (35%)	566 (40%)	
Now smoking	457 (16%)	191 (14%)	266 (18%)	
Drink (*n*, %)				0.188
Never Drinking	158 (5.7%)	70 (5.3%)	88 (6.0%)	
Non-Drink Past 12 Mon	648 (20%)	279 (18%)	369 (23%)	
≤1 Drink/Mon	533 (20%)	240 (21%)	293 (19%)	
>1 Drink/Mon	1,130 (54%)	537 (56%)	593 (53%)	
BMI (*n*, %)				**<0.001**
Normal	611 (23%)	162 (12%)	449 (32%)	
Underweight	34 (0.8%)	4 (0.1%)	30 (1.4%)	
Overweight	1,038 (42%)	467 (42%)	571 (42%)	
Obesity	786 (35%)	493 (46%)	293 (25%)	
Sedentary time (*n*, %)				0.400
< 8 h	1,740 (66%)	798 (67%)	942 (65%)	
≥8 h	729 (34%)	328 (33%)	401 (35%)	
Diabetes (*n*, %)				0.674
Healthy	1,885 (80%)	844 (79%)	1,041 (81%)	
Prediabetes	89 (3.1%)	46 (3.4%)	43 (2.9%)	
Diabetes	495 (17%)	236 (18%)	259 (16%)	
Hypertension (*n*, %)				**0.011**
Healthy	1,288 (55%)	551 (52%)	737 (58%)	
Hypertension	1,181 (45%)	575 (48%)	606 (42%)	
Family history of osteoporosis (Yes, *n*, %)	238 (11%)	97 (8.3%)	141 (14%)	**0.001**
History of prednisone or cortisone use (Yes, *n*, %)	116 (5.3%)	42 (4.1%)	74 (6.2%)	0.103
VD (mcg/d)	7.5 (3.1, 18.0)	6.9 (3.1, 16.8)	7.9 (2.9, 19.1)	0.585
VC (mg/d)	106.7 (45.7, 189.9)	107.8 (41.0, 174.1)	104.7 (50.7, 198.9)	0.248
Calcium (mg/d)	1,022.0 (697.2, 1,402.8)	1,033.7 (716.2, 1,352.5)	1,006.9 (688.0, 1,422.5)	0.770
Phosphorus (mg/d)	1,432.0 (1,117.6, 1,764.8)	1,439.5 (1,140.0, 1,758.7)	1,424.5 (1,100.8, 1,777.0)	0.289
Sodium (mg/d)	3,507.0 (2,739.9, 4,479.7)	3,505.4 (2,715.2, 4,503.5)	3,508.8 (2,750.0, 4,407.3)	0.805
Energy(kcal/d)	2,176.6 (1,728.7, 2,700.3)	2,217.5 (1,757.2, 2,704.6)	2,147.8 (1,693.4, 2,697.8)	0.152
Protein (g/d)	84.9 (64.4, 107.9)	86.1 (65.8, 109.4)	83.3 (63.3, 106.1)	0.169
Caffeine (mg/d)	171.5 (68.5, 294.9)	173.0 (65.5, 297.9)	170.0 (70.2, 294.0)	0.812
VK (mcg/d)	95.2 (59.5, 163.7)	94.5 (60.5, 161.7)	97.3 (58.8, 164.6)	0.871
Level of VK intake (*n*, %)				0.716
Low	1,008 (33%)	462 (32%)	546 (33%)	
Medium	760 (33%)	350 (34%)	410 (32%)	
High	701 (34%)	314 (33%)	387 (35%)	
Women				
N (*n*, %)	2,606 (100%)	722 (27%)	1884 (73%)	
Age (*n*, %)				**<0.001**
50–59 years	1,016 (48%)	383 (61%)	633 (43%)	
60–69 years	950 (31%)	244 (28%)	706 (32%)	
70–79 years	447 (15%)	81 (9.7%)	366 (17%)	
80+ years	193 (6.1%)	14 (1.3%)	179 (7.9%)	
Race (*n*, %)				**<0.001**
NON-Hispanic White	1,210 (73%)	280 (70%)	930 (74%)	
NON-Hispanic Black	528 (9.6%)	245 (17%)	283 (7.0%)	
Mexican American	349 (5.1%)	94 (5.3%)	255 (5.0%)	
Other Hispanic	275 (4.8%)	62 (3.3%)	213 (5.4%)	
Other Race/Multiracial	244 (7.4%)	41 (5.0%)	203 (8.3%)	
Education level (*n*, %)				0.980
Less than college	1,202 (39%)	302 (39%)	900 (39%)	
College or above	1,404 (61%)	420 (61%)	984 (61%)	
Income level (*n*, %)				**0.014**
Low income	696 (17%)	171 (14%)	525 (18%)	
Middle income	1,001 (33%)	274 (29%)	727 (34%)	
High income	909 (50%)	277 (57%)	632 (48%)	
Smoke (*n*, %)				0.104
Never smoking	1,621 (60%)	440 (60%)	1,181 (60%)	
Used smoking	630 (26%)	189 (29%)	441 (25%)	
Now smoking	355 (14%)	93 (11%)	262 (15%)	
Drink (*n*, %)				0.078
Never drinking	519 (15%)	116 (13%)	403 (15%)	
Non-drink past 12 mon	590 (19%)	156 (16%)	434 (20%)	
≤1 Drink/Mon	768 (31%)	226 (31%)	542 (31%)	
>1 Drink/Mon	729 (36%)	224 (40%)	505 (34%)	
BMI (*n*, %)				**<0.001**
Normal	701 (30%)	77 (13%)	624 (36%)	
Underweight	48 (1.9%)	1 (<0.1%)	47 (2.6%)	
Overweight	901 (35%)	218 (32%)	683 (36%)	
Obesity	956 (33%)	426 (55%)	530 (25%)	
Sedentary time (*n*, %)				0.839
< 8 h	1,866 (67%)	506 (67%)	1,360 (67%)	
≥8 h	740 (33%)	216 (33%)	524 (33%)	
Diabetes (*n*, %)				**0.009**
Healthy	2,103 (86%)	555 (81%)	1,548 (88%)	
Prediabetes	76 (2.9%)	28 (4.7%)	48 (2.2%)	
Diabetes	427 (11%)	139 (15%)	288 (10%)	
Hypertension (*n*, %)				0.217
Healthy	1,267 (57%)	327 (53%)	940 (58%)	
Hypertension	1,339 (43%)	395 (47%)	944 (42%)	
Family history of osteoporosis (Yes, *n*, %)	468 (23%)	105 (16%)	363 (26%)	**<0.001**
History of prednisone or cortisone use (Yes, *n*, %)	201 (7.3%)	43 (5.5%)	158 (8.0%)	0.126
History of estrogen use (Yes, *n*, %)	899 (39%)	229 (35%)	670 (40%)	0.104
VD (mcg/d)	12.9 (3.5, 29.7)	12.2 (3.5, 28.5)	13.1 (3.5, 30.9)	0.361
VC (mg/d)	108.1 (48.3, 210.3)	107.3 (53.5, 220.0)	108.2 (45.1, 204.6)	0.385
Calcium (mg/d)	1,034.1 (687.0, 1,516.1)	1,007.4 (687.6, 1,449.2)	1,043.2 (682.1, 1,539.1)	0.496
Phosphorus (mg/d)	1,125.0 (885.1, 1,404.8)	1,188.3 (907.9, 1,483.8)	1,105.3 (873.6, 1,366.6)	**0.014**
Sodium (mg/d)	2,692.4 (2,091.3, 3,398.6)	2,898.2 (2,187.5, 3,675.0)	2,631.5 (2,049.5, 3,286.0)	**0.002**
Energy (kcal/d)	1,653.4 (1,346.2, 2,061.0)	1,718.0 (1,425.4, 2,122.4)	1,630.1 (1,317.0, 2,029.1)	**0.006**
Protein (g/d)	64.0 (50.9, 81.4)	68.6 (53.7, 86.7)	62.9 (50.5, 78.5)	**0.004**
Caffeine (mg/d)	129.5 (50.0, 234.8)	144.9 (48.0, 249.0)	124.6 (51.0, 224.4)	0.153
VK (mcg/d)	92.7 (54.8, 161.0)	99.3 (63.2, 151.0)	90.2 (52.0, 168.5)	0.190
Level of VK intake (*n*, %)				**0.039**
Low	1,036 (33%)	268 (27%)	768 (35%)	
Medium	798 (33%)	250 (39%)	548 (31%)	
High	772 (34%)	204 (34%)	568 (34%)	

^1^Median (P25, P75) for continuous; *n* (%) for categorical. ^2^Chi-squared test with Rao and Scott’s second-order correction; Wilcoxon rank-sum test for complex survey samples. BMI, body mass index; VD, vitamin D; VC, vitamin C; VK, vitamin K.

#### 3.1.1 Osteoporosis and nonosteoporosis groups

The nonosteoporosis group had a younger age, a higher proportion of men, better education levels, a higher percentage of high-income earners, a higher proportion of participants with overweight and obesity, longer sedentary period, and a higher intake of phosphorus, sodium, energy, protein, caffeine, and VK (*p* < 0.05) (Table 1). In contrast, the osteoporosis group had a higher proportion of never-smokers and nonalcohol drinkers; a higher proportion of participants with a family history of osteoporosis and a history of estrogen or glucocorticoid drugs use, and a higher intake of VD (*p* < 0.05) (Table 1). Among men, the nonosteoporosis group was younger and more likely to be overweight or obese, to have never drink alcohol, to have a family history of osteoporosis, and to use glucocorticoids at a higher proportion (*p* < 0.05) (Table 1). The remaining factors did not differ between the groups (*p* > 0.05) (Table 1). In contrast, among women, the between-group differences for the remaining indicators were consistent with the overall results. However, the smoking history, a history of estrogen use, and the intake of VD, VC, calcium, and phosphorus did not differ between the groups (*p* > 0.05). The proportion of women with overweight was lower in the nonosteoporosis group than in the osteoporosis group (*p* < 0.05) (Table 1).

#### 3.1.2 Bone loss and healthy groups

The healthy group had a younger age, a higher proportion of men, a higher proportion of high-income earners, a higher proportion of participants with obesity, and higher intakes of phosphorus, sodium, energy, and protein (*p* < 0.05) (Table 2). In contrast, the bone loss group had a higher proportion of nonalcohol drinkers, a higher percentage of participants without diabetes and hypertension, a higher proportion of participants with a family history of osteoporosis, and higher proportions of estrogen or glucocorticosteroid use (*p* < 0.05) (Table 2). VK intake levels did not differ between the groups (*p* > 0.05) (Table 2). Among men, the healthy group was younger and more likely to be nonsmokers and have obesity, but the osteoporosis group was more likely to be without hypertension and have a family history of osteoporosis (*p* < 0.05) (Table 2). However, VK intake levels did not differ between the groups (*p* > 0.05) (Table 2). Among women, the healthy group was younger and more likely to have a high income, obesity, and a greater intake of phosphorus, sodium, energy, and protein, and a medium-level VK intake (*p* < 0.05) (Table 2). In contrast, the osteoporosis group was more likely to be without diabetes and have a family history of osteoporosis (*p* < 0.05) (Table 2).

### 3.2 Association between VK intake levels and bone health

#### 3.2.1 Association between VK intake levels and bone health among all participants

**Figure 2:**
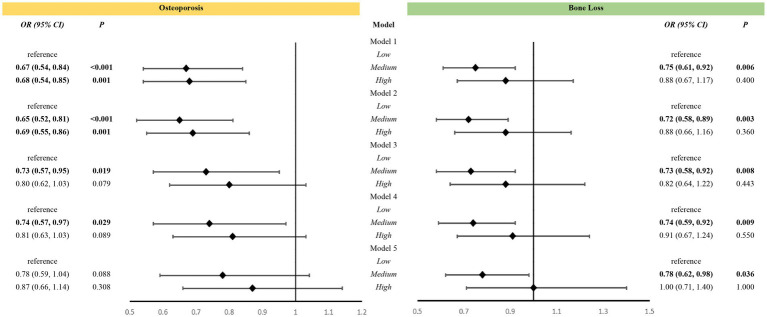
Association between vitamin K intake levels and bone health among all participants. Models: Model 1 was an unadjusted model; Model 2 was adjusted for age and race; Model 3 was further adjusted for education, poverty, BMI, sedentary behavior, history of smoking, and history of alcohol use based on Model 2; Model 4 included Model 3 and was further adjusted for family history of osteoporosis, and history of cortisone use, diabetes mellitus, and hypertension, and for estrogen use in women; Model 5 added dietary variables, including intake of vitamin D, vitamin C, calcium, phosphorus, sodium, energy, protein, and caffeine, to Model 4. Levels: LOW level: <65.95 mcg/d for women, <69.85 mcg/d for men; Medium level: 265.95 mcg/d and <129.40 mcg/d for women, 269.85 mcg/d and <128.75 mcg/d for men; High level: 2129.40 mcg/d for women, 2128.75 mcg/d for men.

High-level VK intake was associated with a reduced risk of osteoporosis in Models 1 and 2. After adjustment for Models 3 and 4, medium-level VK intake was associated with a reduced risk of osteoporosis. However, this association was not observed in Model 5. Nevertheless, medium-level VK intake was associated with a lower risk of bone loss in all five models (Figure 2).

#### 3.2.2 Sex-specific association between VK intake levels and osteoporosis

**Figure 3:**
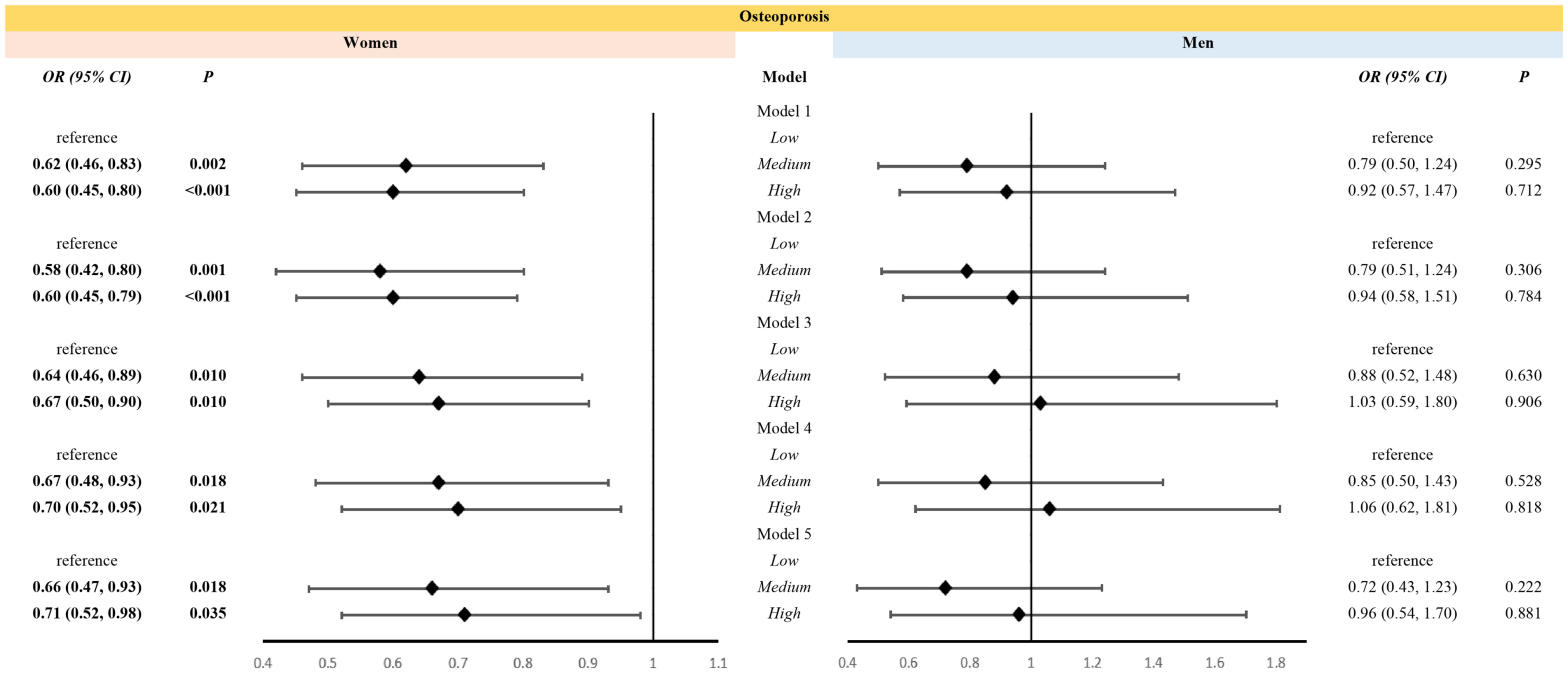
Association between levels of vitamin K intake and osteoporosis in different sexes. Models: Model 1 was an unadjusted model; Model 2 was adjusted for age and race; Model 3 was further adjusted for education, poverty, BMI, sedentary behavior, history of smoking, and history of alcohol use based on Model 2; Model 4 included Model 3 and was further adjusted for family history of osteoporosis, and history of cortisone use, diabetes mellitus, and hypertension, and for estrogen use in women; Model 5 added dietary variables, including intake of vitamin D, vitamin C, calcium, phosphorus, sodium, energy, protein, and caffeine, to Model 4. Levels: LOW level: <65.95 mcg/d for women, <69.85 mcg/d for men; Medium level: 265.95 mcg/d and <129.40 mcg/d for women, 269.85 mcg/d and <128.75 mcg/d for men; High level: 2129.40 mcg/d for women, 2128.75 mcg/d for men.

Weighted multiple logistic regression suggested that medium- and high-level VK intakes were associated with a reduced risk of osteoporosis in women aged over 50 years even after adjustment for covariates. In Model 5, women with medium- [odds ratio, OR (95% confidence interval, CI): 0.66(0.47, 0.93)] and high-level [OR (95% CI): 0.71(0.52, 0.98)] VK intake demonstrated a reduced risk of osteoporosis than women with low-level VK intake (Figure 3). However, VK intake was not associated with osteoporosis in men aged over 50 years (Figure 3).

#### 3.2.3 Sex-specific association between VK intake levels and bone loss

**Figure 4:**
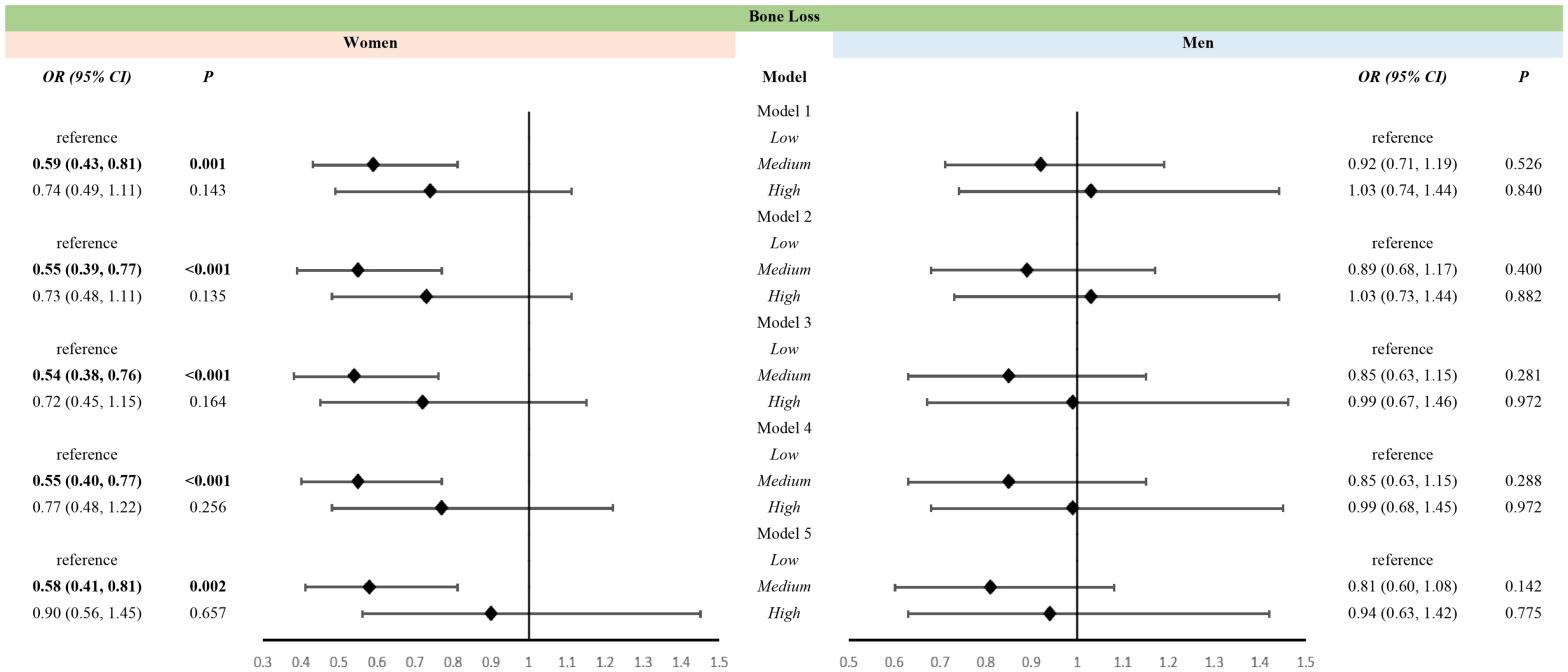
Association between levels of vitamin K intake and bone loss in different sexes. Models: Model 1 was an unadjusted model; Model 2 was adjusted for age and race; Model 3 was further adjusted for education, poverty, BMI, sedentary behavior, history of smoking, and history of alcohol use based on Model 2; Model 4 included Model 3 and was further adjusted for family history of osteoporosis, and history of cortisone use, diabetes mellitus, and hypertension, and for estrogen use in women; Model 5 added dietary variables, including intake of Vitamin D, Vitamin C, calcium, phosphorus, sodium, energy, protein, and caffeine, to Model 4. Levels: Low level: <65.95 mcg/d for women, <69.85 mcg/d for men; Medium level: 265.95 mcg/d and <129.40 mcg/d for women, 269.85 mcg/d and <128.75 mcg/d for men; High level: 2129.40 mcg/d for women, 2128.75 mcg/d for men.

Weighted multiple logistic regression suggested that medium-level VK intake was associated with a reduced risk of bone loss in women aged over 50 years in all five models [OR (95% CI): 0.59(0.43, 0.81); 0.55(0.39, 0.77); 0.54(0.38, 0.76); 0.55(0.40, 0.77); 0.58(0.41, 0.81)] (Figure 4). In contrast, high-level VK intake was not associated with bone loss (Figure 4). Moreover, VK intake was not associated with bone loss in men aged over 50 years (Figure 4).

#### 3.2.4 Site-specific association between VK levels and osteoporosis

For all participants, high-level VK intake was associated with a reduced risk of osteoporosis at all bone sites. Upon considering only VK intake levels with age and race, medium-level VK intake was associated with a reduced risk of osteoporosis at the lumbar spine, femoral neck, intertrochanter, and total femur; however, these associations disappeared upon adjusting all variables (Supplementary Table C). Among women, medium-level VK intake was associated with a reduced risk of osteoporosis at the lumbar spine [OR (95% CI): 0.70(0.49, 0.99)] and high-level VK intake was at the femoral neck [OR (95% CI): 0.67(0.45, 1.00)], respectively, even after adjusted for all variables (Supplementary Table C). In Model 4, which excluded dietary intake, high-level VK intake was associated with a reduced risk of osteoporosis at all five sites (Supplementary Table C). In contrast, among men, VK intake levels were not associated with osteoporosis at all sites (Supplementary Table C).

#### 3.2.5 Site-specific association between VK and bone loss

For all participants, medium-level VK intake was associated with a reduced risk of bone loss at all sites in Model 2, which considered only age and race. Moreover, medium-level VK intake was associated with a reduced risk of bone loss at the lumbar spine [OR (95% CI): 0.79(0.63, 0.99)] and femoral neck [OR (95% CI): 0.77(0.59, 1.00)] in Model 5 (Supplementary Table D). Among men, medium-level VK intake was associated with a reduced risk of bone loss at the femoral neck [OR (95% CI): 0.66(0.48, 0.90)]; high-level VK intake was associated with a reduced risk of bone loss at the lumbar spine [OR (95% CI): 0.68(0.47, 0.99)] only in Model 5 (Supplementary Table D). Among women, in Model 5, medium-level VK intake was associated with a reduced risk of bone loss at the lumbar spine [OR (95% CI): 060(0.43, 0.83)], trochanter [OR (95% CI): 0.66(0.48, 0.91)], intertrochanter [OR (95% CI): 0.66(0.50, 086)], and total femur [OR (95% CI): 0.68(0.51, 0.91)]; In contrast, high-level VK intake was associated with a reduced risk of bone loss at the intertrochanter [OR (95% CI): 0.72(0.54, 0.96)] only (Supplementary Table D). In Model 4, high-level VK intake was associated with a reduced risk of bone loss at the lumbar spine [OR (95% CI): 0.70(0.51, 0.96)] and total femur [OR (95% CI): 0.71(0.52, 0.98)] (Supplementary Table D). Additionally, only in unadjusted Model 1 [OR (95% CI): 0.73(0.54, 1.00)] and age- and race-adjusted Model 2 [OR (95% CI): 0.68(0.49, 0.95)], medium-level VK intake was associated with a reduced risk of bone loss at the femoral neck in women (Supplementary Table D).

## 4 Discussion

This study describes the associations between VK intake levels and the risk of osteoporosis and bone loss in people aged over 50 years. Higher levels of VK intake may be associated with a reduced risk of osteoporosis, whereas only medium-level VK intake was associated with a reduced risk of bone loss in all participants. The associations between VK intake levels and both osteoporosis and bone loss were consistent in women and all participants. However, VK intake levels were not associated with osteoporosis in men, with only medium and high levels of VK intake associated with a reduced risk of bone loss at the femoral neck and lumbar spine, respectively.

To the best of our knowledge, this is the first study to examine the association of VK intake levels with sex and bone health (both osteoporosis and bone loss) in a nationally representative sample. Most studies have focused on the association between VK supplementation and BMD, without considering the total VK intake; they have primarily focused on women. A 3-year-long randomized controlled trial suggested that VK_2_ reduced the rate of decline in BMD at the femoral neck and lumbar spine in postmenopausal women, similar to the current findings (39). A meta-analysis reported that VK_2_ supplementation exerted a protective effect on BMD at the lumbar spine but not at the femoral neck in postmenopausal women (40). Low VK intake, particularly low VK_1_ intake, has been associated with an increased risk of hip fracture but not with reduced BMD (41, 42). A double-blind, placebo-controlled trial demonstrated that VK supplementation reduced serum carboxylated osteocalcin but did not affect the BMD at the lumbar spine or proximal femur (27). In summary, the effects of VK differ according to sex and bone site, confirmed by the current results. In this study, medium- and high-level VK intakes were associated with a reduced risk of osteoporosis in all participants and only women; medium-level VK intake was associated with a reduced risk of bone loss. However, among men, only medium-level VK intake was associated with bone loss at the femoral neck; high-level VK intake was associated with bone loss at the lumbar spine.

In this study, the average VK intake was 95.2 mcg/d for men, which is below the intake (120 mcg/d) recommended by the American Dietetic Association. For women, it was 92.7 mcg/d, which meets the recommended intake (90 mcg/d) (32). Upon grouping VK intake levels based on sex and tertiles, medium-level VK intake (men: 69.85–128.75 mcg/d; women: 65.96–129.40 mcg/d) was close to the recommended intake. Regression analysis results suggested that medium-level VK intake was associated with a reduced risk of bone loss and osteoporosis in all participants and women aged over 50 years. High-level VK intake was associated with a reduced risk of osteoporosis in only women. This finding could be attributed to delayed bone loss or moderately increased BMD at different sites because of VK; however, these changes will not reverse the change in bone health status and may only postpone the age of osteoporosis diagnosis (25, 26, 39). In this study, VD intake level was higher in the osteoporosis group than in the non-osteoporosis group, which may be attributed to additional VD supplementation after osteoporosis diagnosis, thus reversing causality. VK intake levels may follow a similar trend, in which patients consume medically prescribed or self-administered VK supplementation after osteoporosis diagnosis, thus increasing VK intake levels in the osteoporosis group. This aspect potentially masks the effects of high-level VK intake on bone health. Additionally, the physiological effects of nutrients may interact with each other, with synergistic or antagonistic effects. The intake of different nutrients may affect the effects of VK on bone health (43). Therefore, dietary factors were considered continuous covariates for analysis. After controlling for these factors and other covariates, VK intake remained associated with bone health. However, it did not exert a causal effect, thus warranting further studies. Meanwhile, uncarboxylated osteocalcin decreases upon increasing VK intake. Nonetheless, the magnitude of the decrease reduces upon increasing the dose of VK intake, suggesting a physiological saturation effect. Therefore, high-level VK intake is not always beneficial, despite the absence of adverse effects. These findings do not highlight the benefits of high-level VK intake (30, 31, 44). Additionally, the current dose recommendation, determined primarily based on maintaining the physiological function of coagulation, is inadequate for promoting bone health, particularly in postmenopausal women. This necessitates additional studies to determine the appropriate intake of different types of VK (43).

Osteoporosis is most commonly observed in older women. In the US, its prevalence is approximately four times higher in women aged over 50 years (28%) than in age-matched men (6.9%). Additionally, a study based on the Framingham Heart Study (1996–2000) reported that low dietary intake of VK is associated with low BMD in women but not in men, similar to the current findings (45). A study in China demonstrated that VK_2_ supplementation reduced bone loss at the femoral neck in postmenopausal women; however, similar results were not observed at the lumbar spine or hip joints in women, or at any sites in men (44). The present study suggested that medium and high levels of VK intake were associated with a reduced risk of osteoporosis and bone loss in women, but only with a reduced risk of bone loss at the femoral neck and lumbar spine in men. In women, osteoporosis is primarily attributed to physiological changes, mostly due to an imbalance between bone formation and bone resorption caused by reduced postmenopausal estrogen. In contrast, osteoporosis in men is more commonly secondary, caused by various factors, such as metabolic diseases, low body weight, alcohol consumption, and low physical activity (18, 46–48). Differences in osteoporosis between men and women may stem from differences in bone biology and morphology (49). During the early decades of life, bone mass increases because of genetic and environmental factors, which determine the risk of osteoporosis (18). Owing to the differences in hormonal regulation and other aspects between sexes, men have higher peak bone mass and larger bone diameters than women (48, 49). Additionally, unlike women whose estrogen deficiency accelerates bone loss, men do not have hypogonadism, maintain stable hormone levels, and lack a phase of accelerated bone loss. In men, bone remodeling remains low at midlife and lower with age than in women (50, 51). Men experience age-related bone loss; however, the amount is smaller, and the primary mechanism is reduced bone formation (47). A decline in testosterone is the key cause of osteoporosis in men, with the slow age-related decline in bone mass associated with a slow decline in androgen levels. Adequate intake of VK appears to promote the complete carboxylation of osteocalcin, partially inhibiting osteoclasts and promoting osteoblasts, thus partially correcting the imbalance in bone remodeling caused by estrogen reduction in women. This phenomenon may explain the varying effects of VK on the risk of osteoporosis and bone loss in both sexes (23, 52, 53).

To the best of our knowledge, this is the first study to demonstrate that medium-level VK intake is associated with a reduced risk of osteoporosis and bone loss in women aged over 50 years, providing further evidence for the sex-specific recommended VK intake level and subsequent studies on its association with bone health. However, this study has several limitations. First, it was a cross-sectional study, which does not provide sufficient evidence for a causal association between VK intake and bone health, thus warranting a long-term prospective study. Second, because of the differences in the indicators in each cycle of the NHANES database, biochemical indicators were not included. Moreover, the mechanisms between VK and bone metabolism cannot be completely demonstrated. Particularly, the varying effects of different VK intake levels on osteoporosis and bone loss in men and women cannot be entirely clarified, thus warranting further research. Third, owing to the limitations of the database, all types of VK were grouped into one for statistical analysis. Hence, the roles of each type of VK in bone health could not be elucidated, thereby necessitating further studies. Finally, the NHANES used the 24-h dietary recall method for dietary surveys. This method is the gold standard for dietary surveys; however, it is difficult to avoid bias, such as recall deviation and estimation error. The NHANES has collected and checked the data through training and supervision to control its quality. Additionally, data of the dietary surveys in NHANES have been proven reliable (54–58).

## 5 Conclusion

Medium-level VK intake (approximately 65–130 mcg/d) was associated with a reduced risk of osteoporosis and bone loss in people aged over 50 years, particularly women, and with a reduced risk of bone loss at the femoral neck in men. High-level VK intake was associated with a reduced risk of osteoporosis only in women and with a reduced risk of bone loss at the lumbar spine in men. Large prospective cohort studies or randomized controlled trials are warranted to elucidate the effects of varying VK intake levels on bone health at different sites based on sex. Additional studies will facilitate determining appropriate VK intake levels for different populations.

## Data Availability

Publicly available datasets were analyzed in this study. This data can be found at: https://www.cdc.gov/nchs/nhanes/index.htm.

## References

[1] Kanis JA Cooper C Rizzoli R Reginster JY. European guidance for the diagnosis and management of osteoporosis in postmenopausal women. Osteoporosis Int. (2019) 30:3–44. doi: 10.1007/s00198-018-4704-5, PMID: 30324412 PMC7026233

[2] Qaseem A Forciea MA McLean RM Denberg TD Barry MJ Cooke M. Treatment of low bone density or osteoporosis to prevent fractures in men and women: a clinical practice guideline update from the American College of Physicians. Ann Intern Med. (2017) 166:818–39. doi: 10.7326/m15-1361 28492856

[3] Ravazzano L Colaianni G Tarakanova A Xiao YB Grano M Libonati F. Multiscale and multidisciplinary analysis of aging processes in bone. NPJ Aging. (2024) 10:156. doi: 10.1038/s41514-024-00156-2 PMC11180112 38879533

[4] Clynes MA Harvey NC Curtis EM Fuggle NR Dennison EM Cooper C. The epidemiology of osteoporosis. Br Med Bull. (2020) 133:105–17. doi: 10.1093/bmb/ldaa005, PMID: 32282039 PMC7115830

[5] Lane NE. Epidemiology, etiology, and diagnosis of osteoporosis. Am J Obstet Gynecol. (2006) 194:S3–S11. doi: 10.1016/j.ajog.2005.08.047 16448873

[6] Praveen AD Aspelund T Ferguson SJ Sigurðsson S Guðnason V Pálsson H. Refracture and mortality risk in the elderly with osteoporotic fractures: the ages-Reykjavik study. Osteoporosis Int. (2024) 35:1231–41. doi: 10.1007/s00198-024-07096-3, PMID: 38658459 PMC11211172

[7] Khandelwal S Lane NE. Osteoporosis: review of etiology, mechanisms, and approach to management in the aging population. Endocrinol Metab Clin N Am. (2023) 52:259–75. doi: 10.1016/j.ecl.2022.10.009 36948779

[8] Kanis JA Norton N Harvey NC Jacobson T Johansson H Lorentzon M. Scope 2021: a new scorecard for osteoporosis in Europe. Arch Osteoporos. (2021) 16:82. doi: 10.1007/s11657-020-00871-9, PMID: 34080059 PMC8172408

[9] Johnell O Kanis JA. An estimate of the worldwide prevalence and disability associated with osteoporotic fractures. Osteoporosis Int. (2006) 17:1726–33. doi: 10.1007/s00198-006-0172-4, PMID: 16983459

[10] Wright NC Looker AC Saag KG Curtis JR Delzell ES Randall S. The recent prevalence of osteoporosis and low bone mass in the United States based on bone mineral density at the femoral neck or lumbar spine. J Bone Miner Res Off Am Soc Bone Miner Res. (2014) 29:2520–6. doi: 10.1002/jbmr.2269 PMC4757905 24771492

[11] LeBoff MS Greenspan SL Insogna KL Lewiecki EM Saag KG Singer AJ. The clinician's guide to prevention and treatment of osteoporosis. Osteoporosis Int. (2022) 33:2049–102. doi: 10.1007/s00198-021-05900-y, PMID: 35478046 PMC9546973

[12] Vásquez E Alam MT Murillo R. Race and ethnic differences in physical activity, osteopenia, and osteoporosis: results from Nhanes 2009–2010, 2013–2014, 2017–2018. Arch Osteoporos. (2023) 19:1356. doi: 10.1007/s11657-023-01356-1, PMID: 38150070

[13] Yıldız Potter İ Rodriguez EK Wu J Nazarian A Vaziri A. An automated vertebrae localization, segmentation, and osteoporotic compression fracture detection pipeline for computed tomographic imaging. J Imaging Inf Med. (2024) 37:2428–43. doi: 10.1007/s10278-024-01135-5, PMID: 38717516 PMC11522205

[14] Lou Y Wang W Wang C Fu R Shang S Kang Y. Clinical features and burden of osteoporotic fractures among the elderly in the USA from 2016 to 2018. Arch Osteoporos. (2022) 17:78. doi: 10.1007/s11657-022-01113-w 35552890

[15] Wu CH Kao IJ Hung WC Lin SC Liu HC Hsieh MH. Economic impact and cost-effectiveness of fracture liaison services: a systematic review of the literature. Osteoporosis Int. (2018) 29:1227–42. doi: 10.1007/s00198-018-4411-2, PMID: 29460102

[16] Mladěnka P Macáková K Kujovská Krčmová L Javorská L Mrštná K Carazo A. Vitamin K - sources, physiological role, kinetics, deficiency, detection, therapeutic use, and toxicity. Nutr Rev. (2022) 80:677–98. doi: 10.1093/nutrit/nuab061, PMID: 34472618 PMC8907489

[17] Manolagas SC. Birth and death of bone cells: basic regulatory mechanisms and implications for the pathogenesis and treatment of osteoporosis. Endocr Rev. (2000) 21:115–37. doi: 10.1210/edrv.21.2.0395, PMID: 10782361

[18] Vilaca T Eastell R Schini M. Osteoporosis in men. Lancet Diab Endocrinol. (2022) 10:273–83. doi: 10.1016/S2213-8587(22)00012-2 35247315

[19] D'Ippolito G Schiller PC Ricordi C Roos BA Howard GA. Age-related osteogenic potential of mesenchymal stromal stem cells from human vertebral bone marrow. J Bone Miner Res. (1999) 14:1115–22. doi: 10.1359/jbmr.1999.14.7.1115, PMID: 10404011

[20] Weber P. Vitamin K and bone health. Nutrition. (2001) 17:880–7. doi: 10.1016/S0899-9007(01)00709-2 11684396

[21] Hauschka PV Lian JB Cole DE Gundberg CM. Osteocalcin and matrix Gla protein: vitamin K-dependent proteins in bone. Physiol Rev. (1989) 69:990–1047. doi: 10.1152/physrev.1989.69.3.990 2664828

[22] Stock M Schett G. Vitamin K-dependent proteins in skeletal development and disease. Int J Mol Sci. (2021) 22:9328. doi: 10.3390/ijms22179328, PMID: 34502245 PMC8430550

[23] Yamaguchi M Weitzmann MN. Vitamin K2 stimulates osteoblastogenesis and suppresses osteoclastogenesis by suppressing Nf-Κb activation. Int J Mol Med. (2011) 27:3–14. doi: 10.3892/ijmm.2010.562, PMID: 21072493

[24] Palermo A Tuccinardi D D'Onofrio L Watanabe M Maggi D Maurizi AR. Vitamin K and osteoporosis: myth or reality? Metabolism. (2017) 70:57–71. doi: 10.1016/j.metabol.2017.01.032 28403946

[25] Moschonis G Kanellakis S Papaioannou N Schaafsma A Manios Y. Possible site-specific effect of an intervention combining nutrition and lifestyle counselling with consumption of fortified dairy products on bone mass: the postmenopausal health study II. J Bone Miner Metab. (2011) 29:501–6. doi: 10.1007/s00774-010-0256-2, PMID: 21455716

[26] Braam LAJLM Knapen MHJ Geusens P Brouns F Hamulyák K Gerichhausen MJW. Vitamin K1 supplementation retards bone loss in postmenopausal women between 50 and 60 years of age. Calcif Tissue Int. (2003) 73:21–6. doi: 10.1007/s00223-002-2084-4, PMID: 14506950

[27] Binkley N Harke J Krueger D Engelke J Vallarta-Ast N Gemar D. Vitamin K treatment reduces undercarboxylated osteocalcin but does not Alter bone turnover, density, or geometry in healthy postmenopausal north American women. J Bone Miner Res. (2009) 24:983–91. doi: 10.1359/jbmr.081254, PMID: 19113922 PMC2683650

[28] Booth SL Dallal G Shea MK Gundberg C Peterson JW Dawson-Hughes B. Effect of vitamin K supplementation on bone loss in elderly men and women. J Clin Endocrinol Metab. (2008) 93:1217–23. doi: 10.1210/jc.2007-2490 18252784 PMC2291488

[29] Fang Y Hu C Tao X Wan Y Tao F. Effect of vitamin K on bone mineral density: a meta-analysis of randomized controlled trials. J Bone Miner Metab. (2012) 30:60–8. doi: 10.1007/s00774-011-0287-3, PMID: 21674202

[30] K Giri T Newton D Chaudhary O Deych E Napoli N Villareal R. Maximal dose-response of vitamin-K2 (Menaquinone-4) on undercarboxylated osteocalcin in women with osteoporosis. Int J Vitamin Nutr Res. (2020) 90:42–8. doi: 10.1024/0300-9831/a000554, PMID: 30816822

[31] Inaba N Sato T Yamashita T. Low-dose daily intake of vitamin K(2) (Menaquinone-7) improves osteocalcin Γ-carboxylation: a double-blind, randomized controlled trials. J Nutr Sci Vitaminol. (2015) 61:471–80. doi: 10.3177/jnsv.61.471, PMID: 26875489

[32] Storz MA Ronco AL. Nutrient intake in low-carbohydrate diets in comparison to the 2020-2025 dietary guidelines for Americans: a cross-sectional study. Br J Nutr. (2022) 129:1–14. doi: 10.1017/s0007114522001908, PMID: 35730148 PMC9991840

[33] Kanis JA. Assessment of fracture risk and its application to screening for postmenopausal osteoporosis: synopsis of a WHO report. WHO study group. Osteoporosis Int. (1994) 4:368–81. doi: 10.1007/bf01622200, PMID: 7696835

[34] Kanis JA Glüer CC. An update on the diagnosis and assessment of osteoporosis with densitometry. Osteoporosis Int. (2000) 11:192–202. doi: 10.1007/s001980050281 10824234

[35] Binkley N Bilezikian JP Kendler DL Leib ES Lewiecki EM Petak SM. Official positions of the international society for clinical densitometry and executive summary of the 2005 position development conference. J Clin Densitometry. (2006) 9:4–14. doi: 10.1016/j.jocd.2006.05.002, PMID: 16731426

[36] Kanis JA McCloskey EV Johansson H Oden A Melton LJ Khaltaev N. A reference standard for the description of osteoporosis. Bone. (2008) 42:467–75. doi: 10.1016/j.bone.2007.11.001, PMID: 18180210

[37] Centers for Disease Control and Prevention. Weighting module: Centers for Disease Control and Prevention (2024) Available at: https://wwwn.cdc.gov/nchs/nhanes/tutorials/weighting.aspx (Accessed July 11, 2024).

[38] Lachat C Hawwash D Ocké MC Berg C Forsum E Hörnell A. Strengthening the reporting of observational studies in epidemiology - nutritional epidemiology (Strobe-nut): an extension of the Strobe statement. Nutr Bull. (2016) 41:240–51. doi: 10.1111/nbu.12217, PMID: 27587981 PMC4988500

[39] Knapen MHJ Drummen NE Smit E Vermeer C Theuwissen E. Three-year low-dose Menaquinone-7 supplementation helps decrease bone loss in healthy postmenopausal women. Osteoporosis Int. (2013) 24:2499–507. doi: 10.1007/s00198-013-2325-6, PMID: 23525894

[40] Ma ML Ma ZJ He YL Sun H Yang B Ruan BJ. Efficacy of vitamin K2 in the prevention and treatment of postmenopausal osteoporosis: a systematic review and meta-analysis of randomized controlled trials. Front Public Health. (2022) 10:979649. doi: 10.3389/fpubh.2022.979649, PMID: 36033779 PMC9403798

[41] Apalset EM Gjesdal CG Eide GE Tell GS. Intake of vitamin K1 and K2 and risk of hip fractures: the Hordaland health study. Bone. (2011) 49:990–5. doi: 10.1016/j.bone.2011.07.035, PMID: 21839190

[42] Booth SL Tucker KL Chen H Hannan MT Gagnon DR Cupples LA. Dietary vitamin K intakes are associated with hip fracture but not with bone mineral density in elderly men and women. Am J Clin Nutr. (2000) 71:1201–8. doi: 10.1093/ajcn/71.5.1201 10799384

[43] Adams J Pepping J. Vitamin K in the treatment and prevention of osteoporosis and arterial calcification. AJHP. (2005) 62:1574–81. doi: 10.2146/ajhp040357, PMID: 16030366

[44] Zhang Y Liu Z Duan L Ji Y Yang S Zhang Y. Effect of low-dose vitamin K2 supplementation on bone mineral density in middle-aged and elderly Chinese: a randomized controlled study. Calcif Tissue Int. (2020) 106:476–85. doi: 10.1007/s00223-020-00669-4, PMID: 32060566

[45] Booth SL Broe KE Gagnon DR Tucker KL Hannan MT McLean RR. Vitamin K intake and bone mineral density in women and men. Am J Clin Nutr. (2003) 77:512–6. doi: 10.1093/ajcn/77.2.512, PMID: 12540415

[46] Noh J-W Park H Kim M Kwon YD. Gender differences and socioeconomic factors related to osteoporosis: a cross-sectional analysis of nationally representative data. J Womens Health (Larchmt). (2018) 27:196–202. doi: 10.1089/jwh.2016.6244, PMID: 28832241

[47] Seeman E Bianchi G Adami S Kanis J Khosla S Orwoll E. Osteoporosis in men—consensus is premature. Calcif Tissue Int. (2004) 75:120–2. doi: 10.1007/s00223-004-4002-4, PMID: 15185060

[48] Orwig DL Chiles N Jones M Hochberg MC. Osteoporosis in men: update 2011. Rheum Dis Clin North Am. (2011) 37:401–14. doi: 10.1016/j.rdc.2011.08.004, PMID: 22023899

[49] Dy CJ Lamont LE Ton QV Lane JM. Sex and Gender considerations in male patients with osteoporosis. Clin Orthop Relat Res. (2011) 469:1906–12. doi: 10.1007/s11999-011-1849-3, PMID: 21400003 PMC3111783

[50] Orwig DL Chan J Magaziner J. Hip fracture and its consequences: differences between men and women. Orthop Clin N Am. (2006) 37:611–22. doi: 10.1016/j.ocl.2006.08.003 17141019

[51] Bilezikian JP. Osteoporosis in Men*. J Clin Endocrinol Metabol. (1999) 84:3431–4. doi: 10.1210/jcem.84.10.6060 10522975

[52] Cosso R Falchetti A. Vitamin K and bone metabolism: the myth and the truth. Expert Rev Precis Med Drug Dev. (2016) 1:301–17. doi: 10.1080/23808993.2016.1174061

[53] Skalny AV Aschner M Tsatsakis A Rocha JBT Santamaria A Spandidos DA. Role of vitamins beyond vitamin D(3) in bone health and osteoporosis (review). Int J Mol Med. (2024) 53:5333. doi: 10.3892/ijmm.2023.5333 PMC10712697 38063255

[54] Raper N Perloff B Ingwersen L Steinfeldt L Anand J. An overview of USDA's dietary intake data system. J Food Compos Anal. (2004) 17:545–55. doi: 10.1016/j.jfca.2004.02.013

[55] Rhodes DG Murayi T Clemens JC Baer DJ Sebastian RS Moshfegh AJ. The USDA automated multiple-pass method accurately assesses population sodium intakes. Am J Clin Nutr. (2013) 97:958–64. doi: 10.3945/ajcn.112.044982, PMID: 23553153

[56] Rumpler WV Kramer M Rhodes DG Moshfegh AJ Paul DR. Identifying sources of reporting error using measured food intake. Eur J Clin Nutr. (2008) 62:544–52. doi: 10.1038/sj.ejcn.1602742, PMID: 17426745

[57] Blanton CA Moshfegh AJ Baer DJ Kretsch MJ. The USDA automated multiple-pass method accurately estimates group total energy and nutrient intake 12. J Nutr. (2006) 136:2594–9. doi: 10.1093/jn/136.10.2594, PMID: 16988132

[58] Moshfegh AJ Rhodes DG Baer DJ Murayi T Clemens JC Rumpler WV. The US Department of Agriculture automated multiple-pass method reduces bias in the collection of energy intakes. Am J Clin Nutr. (2008) 88:324–32. doi: 10.1093/ajcn/88.2.324, PMID: 18689367

