# Supplementary material for: Vitamin K intake levels are associated with bone health in people aged over 50 years: a NHANES-based survey
Source: Front Med (Lausanne). 2024 Nov 25;11:1485095. doi: 10.3389/fmed.2024.1485095 (PMC11625553; doi:10.3389/fmed.2024.1485095)
Supplement: Supplementary file 1 [file Data_Sheet_1.docx]

***Supplementary Material***

**Supplementary Table A Baseline characteristics of participants in different bone health**

| **Characteristic** | **Overall**^1^ | **Bone Health Groups** | | | ***P***^2^ | ***q***^3^ |
| --- | --- | --- | --- | --- | --- | --- |
|  |  | **Healthy**^1^ | **Osteopenia**^1^ | **Osteoporosis**^1^ |  |  |
| **ALL PARTICIPANTS** | | | | | | |
| **N (n, %)** | 5075 (100%) | 1848 (36%) | 2226 (46%) | 1001 (18%) |  |  |
| **AGE (n, %)** |  |  |  |  | **<0.001** | **<0.001** |
| *50-59 years* | 2,011 (50%) | 912 (61%) | 848 (49%) | 251 (31%) |  |  |
| *60-69 years* | 1,837 (30%) | 652 (28%) | 829 (31%) | 356 (33%) |  |  |
| *70-79 years* | 873 (14%) | 224 (9.0%) | 399 (15%) | 250 (24%) |  |  |
| *80+ years* | 354 (5.2%) | 60 (2.0%) | 150 (4.9%) | 144 (12%) |  |  |
| **GENDER (n, %)** |  |  |  |  | **<0.001** | **<0.001** |
| *Men* | 2,469 (45%) | 1,126 (59%) | 1,150 (46%) | 193 (17%) |  |  |
| *Women* | 2,606 (55%) | 722 (41%) | 1,076 (54%) | 808 (83%) |  |  |
| **RACE (n, %)** |  |  |  |  | **<0.001** | **<0.001** |
| *NON-Hispanic White* | 2,316 (73%) | 719 (70%) | 1,088 (76%) | 509 (74%) |  |  |
| *NON-Hispanic Black* | 1,072 (9.7%) | 571 (15%) | 369 (7.0%) | 132 (6.6%) |  |  |
| *Mexican American* | 705 (5.4%) | 262 (5.6%) | 302 (5.2%) | 141 (5.6%) |  |  |
| *Other Hispanic* | 524 (4.6%) | 178 (3.7%) | 242 (5.0%) | 104 (5.1%) |  |  |
| *Other Race/Multiracial* | 458 (7.0%) | 118 (5.7%) | 225 (7.2%) | 115 (9.2%) |  |  |
| **EDUCATION LEVEL (n, %)** |  |  |  |  | **<0.001** | **0.003** |
| *Less than college* | 2,428 (40%) | 864 (41%) | 1,028 (36%) | 536 (47%) |  |  |
| *College or above* | 2,647 (60%) | 984 (59%) | 1,198 (64%) | 465 (53%) |  |  |
| **INCOME LEVEL (n, %)** |  |  |  |  | **<0.001** | **<0.001** |
| *Low Income* | 1,337 (16%) | 450 (15%) | 572 (15%) | 315 (23%) |  |  |
| *Middle Income* | 1,910 (31%) | 673 (29%) | 847 (31%) | 390 (36%) |  |  |
| *High Income* | 1,828 (52%) | 725 (56%) | 807 (54%) | 296 (41%) |  |  |
| **SMOKE (n, %)** |  |  |  |  | **0.004** | 0.095 |
| *Never Smoking* | 2,658 (54%) | 966 (55%) | 1,117 (51%) | 575 (58%) |  |  |
| *Used Smoking* | 1,605 (32%) | 598 (33%) | 751 (33%) | 256 (24%) |  |  |
| *Now Smoking* | 812 (15%) | 284 (12%) | 358 (16%) | 170 (17%) |  |  |
| **DRINK (n, %)** |  |  |  |  | **<0.001** | **<0.001** |
| *Never Drinking* | 677 (11%) | 186 (8.6%) | 275 (9.9%) | 216 (16%) |  |  |
| *Non-Drink Past 12 Mon* | 1,238 (19%) | 435 (17%) | 537 (18%) | 266 (27%) |  |  |
| *<=1 Drink/Mon* | 1,301 (26%) | 466 (25%) | 567 (26%) | 268 (26%) |  |  |
| *>1 Drink/Mon* | 1,859 (44%) | 761 (50%) | 847 (46%) | 251 (31%) |  |  |
| **BMI (n, %)** |  |  |  |  | **<0.001** | **<0.001** |
| *Normal* | 1,312 (27%) | 239 (12%) | 677 (32%) | 396 (40%) |  |  |
| *Underweight* | 82 (1.4%) | 5 (0.1%) | 38 (1.2%) | 39 (4.4%) |  |  |
| *Overweight* | 1,939 (38%) | 685 (38%) | 904 (39%) | 350 (36%) |  |  |
| *Obesity* | 1,742 (34%) | 919 (50%) | 607 (27%) | 216 (19%) |  |  |
| **SEDENTARY TIME (n, %)** |  |  |  |  | 0.067 | >0.999 |
| *< 8h* | 3,606 (66%) | 1,304 (67%) | 1,573 (64%) | 729 (71%) |  |  |
| *>= 8h* | 1,469 (34%) | 544 (33%) | 653 (36%) | 272 (29%) |  |  |
| **DIABETES (n, %)** |  |  |  |  | **0.010** | 0.238 |
| *Healthy* | 3,988 (83%) | 1,399 (80%) | 1,787 (86%) | 802 (83%) |  |  |
| *Prediabetes* | 165 (3.0%) | 74 (4.0%) | 64 (2.5%) | 27 (2.4%) |  |  |
| *Diabetes* | 922 (14%) | 375 (16%) | 375 (12%) | 172 (14%) |  |  |
| **HYPERTENSION (n, %)** |  |  |  |  | **0.008** | 0.191 |
| *Healthy* | 2,555 (56%) | 878 (52%) | 1,191 (59%) | 486 (54%) |  |  |
| *Hypetension* | 2,520 (44%) | 970 (48%) | 1,035 (41%) | 515 (46%) |  |  |
| **FAMILY HISTORY OF OSTEOPOROSIS (Yes, n, %)** | 706 (18%) | 202 (12%) | 285 (19%) | 219 (28%) | **<0.001** | **<0.001** |
| **HISTORY OF PREDNISONE OR CORTISONE USE (Yes, n, %)** | 317 (6.4%) | 85 (4.7%) | 128 (5.9%) | 104 (11%) | **<0.001** | **<0.001** |
| **HISTORY OF ESTROGEN USE (Yes, n, %)** | 899 (21%) | 229 (15%) | 392 (22%) | 278 (31%) | **<0.001** | **<0.001** |
| **VD (mcg/d)** | 9.7 (3.2, 26.0) | 8.0 (3.2, 21.8) | 9.4 (3.1, 25.6) | 13.7 (3.7, 31.6) | **<0.001** | **0.023** |
| **VC (mg/d)** | 107.4 (46.9, 198.0) | 107.8 (46.6, 185.1) | 104.7 (50.2, 199.5) | 111.3 (41.3, 212.0) | 0.873 | >0.999 |
| **CALCIUM (mg/d)** | 1,027.9 (688.0, 1,454.8) | 1,028.5 (693.3, 1,406.3) | 1,027.7 (675.2, 1,460.7) | 1,017.2 (723.7, 1,562.5) | 0.291 | >0.999 |
| **PHOSPHORUS (mg/d)** | 1,253.0 (959.0, 1,575.5) | 1,346.9 (1,021.5, 1,659.5) | 1,248.6 (955.1, 1,572.2) | 1,114.9 (885.9, 1,417.2) | **<0.001** | **<0.001** |
| **SODIUM (mg/d)** | 3,011.5 (2,300.8, 3,929.9) | 3,211.0 (2,520.8, 4,200.3) | 3,040.1 (2,297.7, 3,904.5) | 2,659.2 (2,051.6, 3,412.7) | **<0.001** | **<0.001** |
| **ENERGY(kcal/d)** | 1,863.5 (1,466.3, 2,384.1) | 2,011.0 (1,575.0, 2,506.1) | 1,858.5 (1,445.8, 2,364.7) | 1,673.8 (1,334.5, 2,086.4) | **<0.001** | **<0.001** |
| **PROTEIN (g/d)** | 73.1 (56.3, 94.0) | 78.3 (60.0, 98.3) | 72.4 (56.0, 93.5) | 63.9 (50.3, 79.8) | **<0.001** | **<0.001** |
| **CAFFEINE (mg/d)** | 145.5 (57.5, 257.0) | 156.0 (57.1, 272.0) | 153.5 (65.0, 259.0) | 107.5 (37.0, 228.0) | **<0.001** | **0.002** |
| **VK (mcg/d)** | 93.8 (57.1, 162.8) | 96.1 (62.5, 157.2) | 96.6 (57.1, 173.7) | 87.2 (51.3, 141.0) | **0.003** | 0.062 |
| **LEVEL OF VK INTAKE (n, %)** |  |  |  |  | **0.009** | 0.226 |
| *Low* | 2,044 (33%) | 730 (30%) | 869 (32%) | 445 (40%) |  |  |
| *Medium* | 1,558 (33%) | 600 (36%) | 673 (32%) | 285 (29%) |  |  |
| *High* | 1,473 (34%) | 518 (34%) | 684 (36%) | 271 (30%) |  |  |
| **MEN** | | | | | | |
| **N (n, %)** | 2469 (100%) | 1126 (46%) | 1150 (47%) | 193 (6.9%) |  |  |
| **AGE (n, %)** |  |  |  |  | **<0.001** | **<0.001** |
| *50-59 years* | 995 (53%) | 529 (62%) | 413 (47%) | 53 (39%) |  |  |
| *60-69 years* | 887 (29%) | 408 (27%) | 418 (31%) | 61 (30%) |  |  |
| *70-79 years* | 426 (13%) | 143 (8.5%) | 238 (17%) | 45 (20%) |  |  |
| *80+ years* | 161 (4.1%) | 46 (2.5%) | 81 (4.7%) | 34 (11%) |  |  |
| **RACE (n, %)** |  |  |  |  | **<0.001** | **0.013** |
| *NON-Hispanic White* | 1,106 (74%) | 439 (71%) | 567 (76%) | 100 (74%) |  |  |
| *NON-Hispanic Black* | 544 (9.8%) | 326 (13%) | 188 (6.6%) | 30 (7.4%) |  |  |
| *Mexican American* | 356 (5.8%) | 168 (5.9%) | 165 (5.8%) | 23 (5.8%) |  |  |
| *Other Hispanic* | 249 (4.3%) | 116 (4.0%) | 116 (4.6%) | 17 (3.2%) |  |  |
| *Other Race/Multiracial* | 214 (6.6%) | 77 (6.2%) | 114 (6.5%) | 23 (9.4%) |  |  |
| **EDUCATION LEVEL (n, %)** |  |  |  |  | 0.364 | >0.999 |
| *Less than college* | 1,226 (41%) | 562 (42%) | 565 (39%) | 99 (46%) |  |  |
| *College or above* | 1,243 (59%) | 564 (58%) | 585 (61%) | 94 (54%) |  |  |
| **INCOME LEVEL (n, %)** |  |  |  |  | 0.473 | >0.999 |
| *Low Income* | 641 (16%) | 279 (16%) | 298 (15%) | 64 (22%) |  |  |
| *Middle Income* | 909 (29%) | 399 (29%) | 445 (30%) | 65 (25%) |  |  |
| *High Income* | 919 (55%) | 448 (56%) | 407 (55%) | 64 (53%) |  |  |
| **SMOKE (n, %)** |  |  |  |  | **0.016** | 0.524 |
| *Never Smoking* | 1,037 (46%) | 526 (51%) | 438 (42%) | 73 (44%) |  |  |
| *Used Smoking* | 975 (38%) | 409 (35%) | 495 (41%) | 71 (33%) |  |  |
| *Now Smoking* | 457 (16%) | 191 (14%) | 217 (17%) | 49 (22%) |  |  |
| **DRINK (n, %)** |  |  |  |  | **0.022** | 0.698 |
| *Never Drinking* | 158 (5.7%) | 70 (5.3%) | 72 (5.4%) | 16 (10.0%) |  |  |
| *Non-Drink Past 12 Mon* | 648 (20%) | 279 (18%) | 308 (21%) | 61 (34%) |  |  |
| *<=1 Drink/Mon* | 533 (20%) | 240 (21%) | 250 (19%) | 43 (18%) |  |  |
| *>1 Drink/Mon* | 1,130 (54%) | 537 (56%) | 520 (55%) | 73 (38%) |  |  |
| **BMI (n, %)** |  |  |  |  | **<0.001** | **<0.001** |
| *Normal* | 611 (23%) | 162 (12%) | 351 (29%) | 98 (49%) |  |  |
| *Underweight* | 34 (0.8%) | 4 (0.1%) | 24 (0.9%) | 6 (5.0%) |  |  |
| *Overweight* | 1,038 (42%) | 467 (42%) | 515 (45%) | 56 (26%) |  |  |
| *Obesity* | 786 (35%) | 493 (46%) | 260 (25%) | 33 (20%) |  |  |
| **SEDENTARY TIME (n, %)** |  |  |  |  | 0.595 | >0.999 |
| *< 8h* | 1,740 (66%) | 798 (67%) | 815 (65%) | 127 (62%) |  |  |
| *>= 8h* | 729 (34%) | 328 (33%) | 335 (35%) | 66 (38%) |  |  |
| **DIABETES (n, %)** |  |  |  |  | 0.235 | >0.999 |
| *Healthy* | 1,885 (80%) | 844 (79%) | 898 (82%) | 143 (74%) |  |  |
| *Prediabetes* | 89 (3.1%) | 46 (3.4%) | 38 (3.0%) | 5 (2.2%) |  |  |
| *Diabetes* | 495 (17%) | 236 (18%) | 214 (15%) | 45 (24%) |  |  |
| **HYPERTENSION (n, %)** |  |  |  |  | 0.077 | >0.999 |
| *Healthy* | 1,288 (55%) | 551 (52%) | 635 (58%) | 102 (56%) |  |  |
| *Hypetension* | 1,181 (45%) | 575 (48%) | 515 (42%) | 91 (44%) |  |  |
| **FAMILY HISTORY OF OSTEOPOROSIS (Yes, n, %)** | 238 (11%) | 97 (8.3%) | 111 (13%) | 30 (20%) | **0.001** | **0.042** |
| **HISTORY OF PREDNISONE OR CORTISONE USE (Yes, n, %)** | 116 (5.3%) | 42 (4.1%) | 55 (5.3%) | 19 (12%) | **0.006** | 0.196 |
| **VD (mcg/d)** | 7.5 (3.1, 18.0) | 6.9 (3.1, 16.8) | 7.6 (2.9, 18.1) | 10.3 (3.0, 30.8) | 0.117 | >0.999 |
| **VC (mg/d)** | 106.7 (45.7, 189.9) | 107.8 (41.0, 174.1) | 102.0 (50.2, 197.9) | 129.5 (60.1, 247.0) | 0.153 | >0.999 |
| **CALCIUM (mg/d)** | 1,022.0 (697.2, 1,402.8) | 1,033.7 (716.2, 1,352.5) | 1,000.3 (681.7, 1,402.2) | 1,009.8 (733.4, 1,622.5) | 0.404 | >0.999 |
| **PHOSPHORUS (mg/d)** | 1,432.0 (1,117.6, 1,764.8) | 1,439.5 (1,140.0, 1,758.7) | 1,428.6 (1,106.1, 1,779.5) | 1,385.0 (1,003.2, 1,699.8) | 0.197 | >0.999 |
| **SODIUM (mg/d)** | 3,507.0 (2,739.9, 4,479.7) | 3,505.4 (2,715.2, 4,503.5) | 3,530.5 (2,755.1, 4,387.3) | 3,431.4 (2,688.2, 4,493.7) | 0.853 | >0.999 |
| **ENERGY(kcal/d)** | 2,176.6 (1,728.7, 2,700.3) | 2,217.5 (1,757.2, 2,704.6) | 2,150.2 (1,695.4, 2,693.3) | 2,033.8 (1,658.4, 2,691.6) | 0.275 | >0.999 |
| **PROTEIN (g/d)** | 84.9 (64.4, 107.9) | 86.1 (65.8, 109.4) | 84.1 (64.6, 106.1) | 76.7 (55.4, 108.7) | 0.089 | >0.999 |
| **CAFFEINE (mg/d)** | 171.5 (68.5, 294.9) | 173.0 (65.5, 297.9) | 173.5 (73.5, 295.2) | 119.8 (29.1, 275.3) | 0.130 | >0.999 |
| **VK (mcg/d)** | 95.2 (59.5, 163.7) | 94.5 (60.5, 161.7) | 97.3 (59.1, 164.5) | 92.7 (57.5, 165.8) | 0.983 | >0.999 |
| **LEVEL OF VK INTAKE (n, %)** |  |  |  |  | 0.847 | >0.999 |
| *Low* | 1,008 (33%) | 462 (32%) | 459 (33%) | 87 (36%) |  |  |
| *Medium* | 760 (33%) | 350 (34%) | 359 (33%) | 51 (29%) |  |  |
| *High* | 701 (34%) | 314 (33%) | 332 (35%) | 55 (35%) |  |  |
| **WOMEN** | | | | | | |
| **N (n, %)** | 2606 (100%) | 722 (27%) | 1076 (45%) | 808 (28%) |  |  |
| **AGE (n, %)** |  |  |  |  | **<0.001** | **<0.001** |
| *50-59 years* | 1,016 (48%) | 383 (61%) | 435 (51%) | 198 (29%) |  |  |
| *60-69 years* | 950 (31%) | 244 (28%) | 411 (31%) | 295 (34%) |  |  |
| *70-79 years* | 447 (15%) | 81 (9.7%) | 161 (13%) | 205 (25%) |  |  |
| *80+ years* | 193 (6.1%) | 14 (1.3%) | 69 (5.0%) | 110 (12%) |  |  |
| **RACE (n, %)** |  |  |  |  | **<0.001** | **<0.001** |
| *NON-Hispanic White* | 1,210 (73%) | 280 (70%) | 521 (75%) | 409 (73%) |  |  |
| *NON-Hispanic Black* | 528 (9.6%) | 245 (17%) | 181 (7.4%) | 102 (6.4%) |  |  |
| *Mexican American* | 349 (5.1%) | 94 (5.3%) | 137 (4.6%) | 118 (5.5%) |  |  |
| *Other Hispanic* | 275 (4.8%) | 62 (3.3%) | 126 (5.3%) | 87 (5.4%) |  |  |
| *Other Race/Multiracial* | 244 (7.4%) | 41 (5.0%) | 111 (7.7%) | 92 (9.2%) |  |  |
| **EDUCATION LEVEL (n, %)** |  |  |  |  | **<0.001** | **0.017** |
| *Less than college* | 1,202 (39%) | 302 (39%) | 463 (34%) | 437 (47%) |  |  |
| *College or above* | 1,404 (61%) | 420 (61%) | 613 (66%) | 371 (53%) |  |  |
| **INCOME LEVEL (n, %)** |  |  |  |  | **<0.001** | **<0.001** |
| *Low Income* | 696 (17%) | 171 (14%) | 274 (15%) | 251 (23%) |  |  |
| *Middle Income* | 1,001 (33%) | 274 (29%) | 402 (32%) | 325 (39%) |  |  |
| *High Income* | 909 (50%) | 277 (57%) | 400 (54%) | 232 (38%) |  |  |
| **SMOKE (n, %)** |  |  |  |  | 0.167 | >0.999 |
| *Never Smoking* | 1,621 (60%) | 440 (60%) | 679 (59%) | 502 (61%) |  |  |
| *Used Smoking* | 630 (26%) | 189 (29%) | 256 (27%) | 185 (23%) |  |  |
| *Now Smoking* | 355 (14%) | 93 (11%) | 141 (14%) | 121 (16%) |  |  |
| **DRINK (n, %)** |  |  |  |  | **<0.001** | **0.002** |
| *Never Drinking* | 519 (15%) | 116 (13%) | 203 (14%) | 200 (17%) |  |  |
| *Non-Drink Past 12 Mon* | 590 (19%) | 156 (16%) | 229 (16%) | 205 (26%) |  |  |
| *<=1 Drink/Mon* | 768 (31%) | 226 (31%) | 317 (33%) | 225 (28%) |  |  |
| *>1 Drink/Mon* | 729 (36%) | 224 (40%) | 327 (38%) | 178 (29%) |  |  |
| **BMI (n, %)** |  |  |  |  | **<0.001** | **<0.001** |
| *Normal* | 701 (30%) | 77 (13%) | 326 (35%) | 298 (38%) |  |  |
| *Underweight* | 48 (1.9%) | 1 (<0.1%) | 14 (1.4%) | 33 (4.3%) |  |  |
| *Overweight* | 901 (35%) | 218 (32%) | 389 (34%) | 294 (39%) |  |  |
| *Obesity* | 956 (33%) | 426 (55%) | 347 (29%) | 183 (19%) |  |  |
| **SEDENTARY TIME (n, %)** |  |  |  |  | 0.072 | >0.999 |
| *< 8h* | 1,866 (67%) | 506 (67%) | 758 (64%) | 602 (72%) |  |  |
| *>= 8h* | 740 (33%) | 216 (33%) | 318 (36%) | 206 (28%) |  |  |
| **DIABETES (n, %)** |  |  |  |  | **0.009** | 0.288 |
| *Healthy* | 2,103 (86%) | 555 (81%) | 889 (89%) | 659 (85%) |  |  |
| *Prediabetes* | 76 (2.9%) | 28 (4.7%) | 26 (2.1%) | 22 (2.5%) |  |  |
| *Diabetes* | 427 (11%) | 139 (15%) | 161 (8.8%) | 127 (12%) |  |  |
| **HYPERTENSION (n, %)** |  |  |  |  | 0.073 | >0.999 |
| *Healthy* | 1,267 (57%) | 327 (53%) | 556 (60%) | 384 (54%) |  |  |
| *Hypetension* | 1,339 (43%) | 395 (47%) | 520 (40%) | 424 (46%) |  |  |
| **FAMILY HISTORY OF OSTEOPOROSIS (Yes, n, %)** | 468 (23%) | 105 (16%) | 174 (23%) | 189 (29%) | **<0.001** | **0.019** |
| **HISTORY OF PREDNISONE OR CORTISONE USE (Yes, n, %)** | 201 (7.3%) | 43 (5.5%) | 73 (6.4%) | 85 (11%) | **0.009** | 0.294 |
| **HISTORY OF ESTROGEN USE (Yes, n, %)** | 899 (39%) | 229 (35%) | 392 (42%) | 278 (38%) | 0.126 | >0.999 |
| **VD (mcg/d)** | 12.9 (3.5, 29.7) | 12.2 (3.5, 28.5) | 12.1 (3.2, 30.7) | 14.3 (3.8, 31.7) | 0.342 | >0.999 |
| **VC (mg/d)** | 108.1 (48.3, 210.3) | 107.3 (53.5, 220.0) | 107.6 (48.8, 204.8) | 108.3 (40.8, 204.6) | 0.340 | >0.999 |
| **CALCIUM (mg/d)** | 1,034.1 (687.0, 1,516.1) | 1,007.4 (687.6, 1,449.2) | 1,059.8 (666.6, 1,527.9) | 1,027.0 (719.1, 1,550.7) | 0.696 | >0.999 |
| **PHOSPHORUS (mg/d)** | 1,125.0 (885.1, 1,404.8) | 1,188.3 (907.9, 1,483.8) | 1,125.1 (875.1, 1,377.8) | 1,092.6 (868.5, 1,352.6) | **0.021** | 0.663 |
| **SODIUM (mg/d)** | 2,692.4 (2,091.3, 3,398.6) | 2,898.2 (2,187.5, 3,675.0) | 2,671.2 (2,104.2, 3,329.7) | 2,491.7 (1,978.5, 3,214.9) | **<0.001** | **0.015** |
| **ENERGY(kcal/d)** | 1,653.4 (1,346.2, 2,061.0) | 1,718.0 (1,425.4, 2,122.4) | 1,646.3 (1,329.0, 2,060.2) | 1,617.1 (1,307.0, 1,994.0) | **0.006** | 0.196 |
| **PROTEIN (g/d)** | 64.0 (50.9, 81.4) | 68.6 (53.7, 86.7) | 63.6 (50.6, 80.3) | 62.2 (50.1, 76.0) | **0.005** | 0.160 |
| **CAFFEINE (mg/d)** | 129.5 (50.0, 234.8) | 144.9 (48.0, 249.0) | 135.6 (58.6, 233.6) | 104.5 (37.5, 218.3) | **0.023** | 0.763 |
| **VK (mcg/d)** | 92.7 (54.8, 161.0) | 99.3 (63.2, 151.0) | 94.8 (55.2, 183.8) | 83.3 (49.9, 140.7) | **<0.001** | **0.024** |
| **LEVEL OF VK INTAKE (n, %)** |  |  |  |  | **0.006** | 0.209 |
| *Low* | 1,036 (33%) | 268 (27%) | 410 (32%) | 358 (41%) |  |  |
| *Medium* | 798 (33%) | 250 (39%) | 314 (32%) | 234 (29%) |  |  |
| *High* | 772 (34%) | 204 (34%) | 352 (37%) | 216 (30%) |  |  |
| ^1^median (P_25_, P_75_) for continuous; n (%) for categorical.  ^2^chi-squared test with Rao & Scott's second-order correction; Wilcoxon rank-sum test for complex survey samples.  ^3^Bonferroni correction for multiple testing.  BMI: body mass index; VD: vitamin D; VC: vitamin C; VK: vitamin K. | | | | | | |

**Supplementary Table B Baseline characteristics of all participants**

| **Characteristic** | **Overall**,  N = 5075 (100%)^1^ | **Gender** | | ***P* Value**^2^ |
| --- | --- | --- | --- | --- |
|  |  | **Women**,  N = 2606 (55%)^1^ | **Men**,  N = 2469 (45%)^1^ |  |
| **AGE (n, %)** |  |  |  | **0.003** |
| *50-59 years* | 2,011 (50%) | 1,016 (48%) | 995 (53%) |  |
| *60-69 years* | 1,837 (30%) | 950 (31%) | 887 (29%) |  |
| *70-79 years* | 873 (14%) | 447 (15%) | 426 (13%) |  |
| *80+ years* | 354 (5.2%) | 193 (6.1%) | 161 (4.1%) |  |
| **RACE (n, %)** |  |  |  | 0.288 |
| *Non-Hispanic White* | 2,316 (73%) | 1,210 (73%) | 1,106 (74%) |  |
| *Non-Hispanic Black* | 1,072 (9.7%) | 528 (9.6%) | 544 (9.8%) |  |
| *Mexican American* | 705 (5.4%) | 349 (5.1%) | 356 (5.8%) |  |
| *Other Hispanic* | 524 (4.6%) | 275 (4.8%) | 249 (4.3%) |  |
| *Other Race/Multiracial* | 458 (7.0%) | 244 (7.4%) | 214 (6.6%) |  |
| **EDUCATION LEVEL (n, %)** |  |  |  | 0.446 |
| *Less than college* | 2,428 (40%) | 1,202 (39%) | 1,226 (41%) |  |
| *College or above* | 2,647 (60%) | 1,404 (61%) | 1,243 (59%) |  |
| **INCOME LEVEL (n, %)** |  |  |  | **0.021** |
| *Low Income* | 1,337 (16%) | 696 (17%) | 641 (16%) |  |
| *Middle Income* | 1,910 (31%) | 1,001 (33%) | 909 (29%) |  |
| *High Income* | 1,828 (52%) | 909 (50%) | 919 (55%) |  |
| **SMOKE (n, %)** |  |  |  | **<0.001** |
| *Never Smoked* | 2,658 (54%) | 1,621 (60%) | 1,037 (46%) |  |
| *Used Smoked* | 1,605 (32%) | 630 (26%) | 975 (38%) |  |
| *Current smoker* | 812 (15%) | 355 (14%) | 457 (16%) |  |
| **DRINK (n, %)** |  |  |  | **<0.001** |
| *Never Drank* | 677 (11%) | 519 (15%) | 158 (5.7%) |  |
| *Non-Drank Past 12 Mon* | 1,238 (19%) | 590 (19%) | 648 (20%) |  |
| *<=1 Drink per Month* | 1,301 (26%) | 768 (31%) | 533 (20%) |  |
| *>1 Drink per Month* | 1,859 (44%) | 729 (36%) | 1,130 (54%) |  |
| **BMI (n, %)** |  |  |  | **<0.001** |
| *Normal* | 1,312 (27%) | 701 (30%) | 611 (23%) |  |
| *Underweight* | 82 (1.4%) | 48 (1.9%) | 34 (0.8%) |  |
| *Overweight* | 1,939 (38%) | 901 (35%) | 1,038 (42%) |  |
| *Obesity* | 1,742 (34%) | 956 (33%) | 786 (35%) |  |
| **SEDENTARY TIME (n, %)** |  |  |  | 0.615 |
| *< 8 h* | 3,606 (66%) | 1,866 (67%) | 1,740 (66%) |  |
| *>= 8 h* | 1,469 (34%) | 740 (33%) | 729 (34%) |  |
| **Diabetes (n, %)** |  |  |  | **<0.001** |
| *Healthy* | 3,988 (83%) | 2,103 (86%) | 1,885 (80%) |  |
| *Prediabetes* | 165 (3.0%) | 76 (2.9%) | 89 (3.1%) |  |
| *Diabetes* | 922 (14%) | 427 (11%) | 495 (17%) |  |
| **Hypertension (Yes, n, %)** | 2,520 (44%) | 1,339 (43%) | 1,181 (45%) | 0.432 |
| **FAMILY HISTORY OF**  **OSTEOPOROSIS (Yes, n, %)** | 706 (18%) | 468 (23%) | 238 (11%) | **<0.001** |
| **HISTORY OF PREDNISONE**  **OR CORTISONE USE (Yes, n, %)** | 317 (6.4%) | 201 (7.3%) | 116 (5.3%) | **0.033** |
| **HISTORY OF ESTROGEN USE (Yes, n, %)** | 899 (21%) | 899 (39%) | - | **-** |
| **VD (mcg/d)** | 9.7 (3.2, 26.0) | 12.9 (3.5, 29.7) | 7.5 (3.1, 18.0) | **<0.001** |
| **VC (mg/d)** | 107.4 (46.9, 198.0) | 108.1 (48.3, 210.3) | 106.7 (45.7, 189.9) | 0.238 |
| **CALCIUM (mg/d)** | 1,027.9 (688.0, 1,454.8) | 1,034.1 (687.0, 1,516.1) | 1,022.0 (697.2, 1,402.8) | 0.431 |
| **PHOSPHORUS (mg/d)** | 1,253.0 (959.0, 1,575.5) | 1,125.0 (885.1, 1,404.8) | 1,432.0 (1,117.6, 1,764.8) | **<0.001** |
| **SODIUM (mg/d)** | 3,011.5 (2,300.8, 3,929.9) | 2,692.4 (2,091.3, 3,398.6) | 3,507.0 (2,739.9, 4,479.7) | **<0.001** |
| **ENERGY (kcal/d)** | 1,863.5 (1,466.3, 2,384.1) | 1,653.4 (1,346.2, 2,061.0) | 2,176.6 (1,728.7, 2,700.3) | **<0.001** |
| **PROTEIN (g/d)** | 73.1 (56.3, 94.0) | 64.0 (50.9, 81.4) | 84.9 (64.4, 107.9) | **<0.001** |
| **CAFFEINE (mg/d)** | 145.5 (57.5, 257.0) | 129.5 (50.0, 234.8) | 171.5 (68.5, 294.9) | **<0.001** |
| **VK (mcg/d)** | 93.8 (57.1, 162.8) | 92.7 (54.8, 161.0) | 95.2 (59.5, 163.7) | 0.583 |
| **LEVEL OF VK INTAKE (n, %)** |  |  |  | 0.986 |
| *Low* | 2,044 (33%) | 1,036 (33%) | 1,008 (33%) |  |
| *Medium* | 1,558 (33%) | 798 (33%) | 760 (33%) |  |
| *High* | 1,473 (34%) | 772 (34%) | 701 (34%) |  |
| **OVERALL (n, %)** |  |  |  | **<0.001** |
| *Healthy* | 1,848 (36%) | 722 (27%) | 1,126 (46%) |  |
| *Osteopenia* | 2,226 (46%) | 1,076 (45%) | 1,150 (47%) |  |
| *Osteoporosis* | 1,001 (18%) | 808 (28%) | 193 (6.9%) |  |
| **BMD OF FEROMAL NECK (gm/cm^2^)** | 0.75 (0.67, 0.85) | 0.72 (0.63, 0.80) | 0.80 (0.72, 0.89) | **<0.001** |
| **FEROMAL NECK (n, %)** |  |  |  | **<0.001** |
| *Healthy* | 2,440 (47%) | 1,133 (41%) | 1,307 (53%) |  |
| *Osteopenia* | 2,339 (48%) | 1,256 (51%) | 1,083 (44%) |  |
| *Osteoporosis* | 296 (5.6%) | 217 (7.7%) | 79 (2.9%) |  |
| **BMD OF TROCHANTER (gm/cm^2^)** | 0.68 (0.61, 0.78) | 0.64 (0.57, 0.72) | 0.75 (0.66, 0.83) | **<0.001** |
| **TROCHANTER (n, %)** |  |  |  | **<0.001** |
| *Healthy* | 3,443 (67%) | 1,575 (60%) | 1,868 (76%) |  |
| *Osteopenia* | 1,502 (31%) | 924 (36%) | 578 (23%) |  |
| *Osteoporosis* | 130 (2.2%) | 107 (3.3%) | 23 (0.9%) |  |
| **BMD OF INTERTROCHANTER (gm/cm^2^)** | 1.09 (0.97, 1.21) | 1.01 (0.91, 1.13) | 1.17 (1.07, 1.28) | **<0.001** |
| **INTERTROCHANTER (n, %)** |  |  |  | **<0.001** |
| *Healthy* | 3,709 (72%) | 1,723 (65%) | 1,986 (81%) |  |
| *Osteopenia* | 1,235 (25%) | 765 (31%) | 470 (18%) |  |
| *Osteoporosis* | 131 (2.4%) | 118 (4.1%) | 13 (0.4%) |  |
| **BMD OF TOTAL FEMUR (gm/cm^2^)** | 0.91 (0.81, 1.02) | 0.85 (0.76, 0.95) | 0.99 (0.90, 1.08) | **<0.001** |
| **TOTAL FEMUR (n, %)** |  |  |  | **<0.001** |
| *Healthy* | 3,354 (66%) | 1,547 (59%) | 1,807 (75%) |  |
| *Osteopenia* | 1,556 (31%) | 918 (36%) | 638 (25%) |  |
| *Osteoporosis* | 165 (3.0%) | 141 (4.9%) | 24 (0.8%) |  |
| **BMD OF LUMBAR SPINE (gm/cm^2^)** | 0.99 (0.88, 1.10) | 0.94 (0.85, 1.05) | 1.05 (0.95, 1.15) | **<0.001** |
| **LUMBAR SPINE (n, %)** |  |  |  | **<0.001** |
| *Healthy* | 2,975 (59%) | 1,132 (45%) | 1,843 (76%) |  |
| *Osteopenia* | 1,574 (31%) | 1,028 (40%) | 546 (21%) |  |
| *Osteoporosis* | 526 (9.3%) | 446 (15%) | 80 (2.5%) |  |
| ^1^median (P25, P75) for continuous; n (%) for categorical. | | | | |
| ^2^chi-squared test with Rao & Scott's second-order correction; Wilcoxon rank-sum test for complex survey samples.  BMI: body mass index; VD: vitamin D; VC: vitamin C; VK: vitamin K. | | | | |

**Supplementary Table C Association between vitamin K intake levels and osteoporosis at different sites**

|  | Model 1 | | | Model 2 | | | Model 3 | | | Model 4 | | | Model 5 | | |
| --- | --- | --- | --- | --- | --- | --- | --- | --- | --- | --- | --- | --- | --- | --- | --- |
| **Characteristic**^1^ | ***OR***^2^ | **95% *CI***^2^ | ***P*** | ***OR***^2^ | **95% *CI***^2^ | ***P*** | ***OR***^2^ | **95% *CI***^2^ | ***P*** | ***OR***^2^ | **95% *CI***^2^ | ***P*** | ***OR***^2^ | **95% *CI***^2^ | ***P*** |
| **ALL PARTICIPANTS** | | | | | | | | | | | | | | | |
| **LUMBAR SPINE** |  |  |  |  |  |  |  |  |  |  |  |  |  |  |  |
| *Low* | — | — |  | — | — |  | — | — |  | — | — |  | — | — |  |
| *Medium* | 0.71 | 0.55, 0.90 | **0.005** | 0.69 | 0.54, 0.88 | **0.004** | 0.77 | 0.58, 1.01 | 0.058 | 0.77 | 0.58, 1.03 | 0.081 | 0.83 | 0.60, 1.14 | 0.237 |
| *High* | 0.68 | 0.53, 0.89 | **0.006** | 0.70 | 0.54, 0.91 | **0.007** | 0.80 | 0.60, 1.07 | 0.124 | 0.81 | 0.61, 1.07 | 0.128 | 0.89 | 0.64, 1.23 | 0.458 |
| **FEROMAL NECK** |  |  |  |  |  |  |  |  |  |  |  |  |  |  |  |
| *Low* | — | — |  | — | — |  | — | — |  | — | — |  | — | — |  |
| *Medium* | 0.68 | 0.53, 0.88 | **0.004** | 0.66 | 0.50, 0.85 | **0.002** | 0.76 | 0.58, 1.00 | 0.052 | 0.77 | 0.59, 1.02 | 0.064 | 0.85 | 0.63, 1.14 | 0.273 |
| *High* | 0.61 | 0.46, 0.80 | **<0.001** | 0.60 | 0.45, 0.81 | **0.001** | 0.71 | 0.52, 0.98 | **0.038** | 0.72 | 0.53, 0.97 | **0.033** | 0.86 | 0.63, 1.17 | 0.325 |
| **TROCHANTER** |  |  |  |  |  |  |  |  |  |  |  |  |  |  |  |
| *Low* | — | — |  | — | — |  | — | — |  | — | — |  | — | — |  |
| *Medium* | 0.78 | 0.60, 1.02 | 0.074 | 0.77 | 0.58, 1.01 | 0.058 | 0.89 | 0.66, 1.19 | 0.422 | 0.91 | 0.68, 1.22 | 0.516 | 1.00 | 0.72, 1.38 | 0.982 |
| *High* | 0.66 | 0.50, 0.88 | **0.005** | 0.67 | 0.50, 0.90 | **0.009** | 0.80 | 0.59, 1.10 | 0.170 | 0.80 | 0.59, 1.08 | 0.142 | 0.95 | 0.68, 1.32 | 0.748 |
| **INTERTROCHANTER** |  |  |  |  |  |  |  |  |  |  |  |  |  |  |  |
| *Low* | — | — |  | — | — |  | — | — |  | — | — |  | — | — |  |
| *Medium* | 0.74 | 0.56, 0.99 | **0.043** | 0.73 | 0.54, 0.97 | **0.033** | 0.83 | 0.60, 1.14 | 0.243 | 0.84 | 0.61, 1.16 | 0.295 | 0.94 | 0.66, 1.34 | 0.742 |
| *High* | 0.63 | 0.46, 0.85 | **0.003** | 0.63 | 0.46, 0.87 | **0.006** | 0.74 | 0.52, 1.06 | 0.099 | 0.74 | 0.53, 1.04 | 0.079 | 0.91 | 0.64, 1.29 | 0.584 |
| **TOTAL FEMUR** |  |  |  |  |  |  |  |  |  |  |  |  |  |  |  |
| *Low* | — | — |  | — | — |  | — | — |  | — | — |  | — | — |  |
| *Medium* | 0.75 | 0.57, 1.01 | 0.055 | 0.74 | 0.55, 0.99 | **0.042** | 0.86 | 0.63, 1.18 | 0.334 | 0.87 | 0.64, 1.20 | 0.395 | 0.96 | 0.68, 1.37 | 0.826 |
| *High* | 0.62 | 0.46, 0.83 | **0.002** | 0.63 | 0.46, 0.85 | **0.004** | 0.76 | 0.54, 1.05 | 0.095 | 0.75 | 0.55, 1.02 | 0.069 | 0.89 | 0.63, 1.25 | 0.494 |
| **MEN** | | | | | | | | | | | | | | | |
| **LUMBAR SPINE** |  |  |  |  |  |  |  |  |  |  |  |  |  |  |  |
| *Low* | — | — |  | — | — |  | — | — |  | — | — |  | — | — |  |
| *Medium* | 0.96 | 0.49, 1.90 | 0.909 | 0.99 | 0.50, 1.93 | 0.967 | 0.98 | 0.46, 2.09 | 0.955 | 0.95 | 0.45, 2.01 | 0.890 | 0.74 | 0.32, 1.71 | 0.472 |
| *High* | 1.07 | 0.59, 1.95 | 0.815 | 1.12 | 0.61, 2.07 | 0.713 | 1.12 | 0.56, 2.25 | 0.748 | 1.16 | 0.58, 2.30 | 0.673 | 0.89 | 0.42, 1.93 | 0.770 |
| **FEROMAL NECK** |  |  |  |  |  |  |  |  |  |  |  |  |  |  |  |
| *Low* | — | — |  | — | — |  | — | — |  | — | — |  | — | — |  |
| *Medium* | 0.65 | 0.40, 1.06 | 0.081 | 0.64 | 0.40, 1.04 | 0.072 | 0.70 | 0.40, 1.23 | 0.209 | 0.65 | 0.37, 1.15 | 0.137 | 0.65 | 0.35, 1.24 | 0.185 |
| *High* | 0.95 | 0.55, 1.67 | 0.869 | 0.97 | 0.55, 1.69 | 0.903 | 1.04 | 0.53, 2.03 | 0.916 | 1.07 | 0.56, 2.02 | 0.839 | 1.11 | 0.62, 1.98 | 0.728 |
| **TROCHANTER** |  |  |  |  |  |  |  |  |  |  |  |  |  |  |  |
| *Low* | — | — |  | — | — |  | — | — |  | — | — |  | — | — |  |
| *Medium* | 1.01 | 0.42, 2.45 | 0.978 | 1.01 | 0.41, 2.50 | 0.982 | 1.06 | 0.43, 2.61 | 0.901 | 0.93 | 0.39, 2.24 | 0.873 | 0.80 | 0.27, 2.34 | 0.678 |
| *High* | 1.13 | 0.52, 2.47 | 0.759 | 1.19 | 0.53, 2.67 | 0.674 | 1.18 | 0.48, 2.90 | 0.706 | 1.19 | 0.46, 3.07 | 0.711 | 0.91 | 0.37, 2.26 | 0.834 |
| **INTERTROCHANTER** |  |  |  |  |  |  |  |  |  |  |  |  |  |  |  |
| *Low* | — | — |  | — | — |  | — | — |  | — | — |  | — | — |  |
| *Medium* | 1.22 | 0.50, 2.98 | 0.653 | 1.23 | 0.50, 3.01 | 0.653 | 1.25 | 0.49, 3.22 | 0.632 | 1.10 | 0.45, 2.72 | 0.831 | 0.96 | 0.32, 2.85 | 0.940 |
| *High* | 1.40 | 0.62, 3.17 | 0.408 | 1.49 | 0.63, 3.48 | 0.356 | 1.50 | 0.59, 3.84 | 0.390 | 1.54 | 0.60, 3.95 | 0.363 | 1.18 | 0.48, 2.88 | 0.714 |
| **TOTAL FEMUR** |  |  |  |  |  |  |  |  |  |  |  |  |  |  |  |
| *Low* | — | — |  | — | — |  | — | — |  | — | — |  | — | — |  |
| *Medium* | 1.08 | 0.46, 2.54 | 0.861 | 1.08 | 0.46, 2.56 | 0.856 | 1.11 | 0.45, 2.74 | 0.817 | 0.99 | 0.41, 2.36 | 0.979 | 0.86 | 0.30, 2.44 | 0.770 |
| *High* | 1.25 | 0.57, 2.71 | 0.575 | 1.32 | 0.59, 2.97 | 0.498 | 1.34 | 0.55, 3.29 | 0.514 | 1.37 | 0.56, 3.37 | 0.483 | 1.06 | 0.46, 2.45 | 0.882 |
| **WOMEN** | | | | | | | | | | | | | | | |
| **LUMBAR SPINE** |  |  |  |  |  |  |  |  |  |  |  |  |  |  |  |
| *Low* | — | — |  | — | — |  | — | — |  | — | — |  | — | — |  |
| *Medium* | 0.64 | 0.48, 0.87 | **0.005** | 0.61 | 0.44, 0.84 | **0.003** | 0.67 | 0.48, 0.94 | **0.021** | 0.70 | 0.50, 0.97 | **0.035** | 0.70 | 0.49, 0.99 | **0.045** |
| *High* | 0.60 | 0.44, 0.82 | **0.002** | 0.60 | 0.45, 0.82 | **0.001** | 0.68 | 0.49, 0.93 | **0.019** | 0.70 | 0.51, 0.97 | **0.034** | 0.72 | 0.50, 1.04 | 0.077 |
| **FEROMAL NECK** |  |  |  |  |  |  |  |  |  |  |  |  |  |  |  |
| *Low* | — | — |  | — | — |  | — | — |  | — | — |  | — | — |  |
| *Medium* | 0.68 | 0.50, 0.92 | **0.014** | 0.63 | 0.45, 0.88 | **0.008** | 0.72 | 0.51, 1.01 | 0.055 | 0.75 | 0.54, 1.05 | 0.096 | 0.77 | 0.54, 1.10 | 0.151 |
| *High* | 0.52 | 0.38, 0.71 | **<0.001** | 0.50 | 0.35, 0.73 | **<0.001** | 0.58 | 0.40, 0.84 | **0.005** | 0.60 | 0.41, 0.87 | **0.008** | 0.67 | 0.45, 1.00 | **0.049** |
| **TROCHANTER** |  |  |  |  |  |  |  |  |  |  |  |  |  |  |  |
| *Low* | — | — |  | — | — |  | — | — |  | — | — |  | — | — |  |
| *Medium* | 0.74 | 0.55, 1.01 | 0.061 | 0.71 | 0.51, 0.99 | **0.043** | 0.82 | 0.58, 1.15 | 0.238 | 0.86 | 0.61, 1.20 | 0.364 | 0.87 | 0.61, 1.25 | 0.438 |
| *High* | 0.59 | 0.43, 0.81 | **0.001** | 0.58 | 0.41, 0.83 | **0.003** | 0.68 | 0.49, 0.96 | **0.027** | 0.70 | 0.50, 0.98 | **0.037** | 0.77 | 0.52, 1.13 | 0.168 |
| **INTERTROCHANTER** |  |  |  |  |  |  |  |  |  |  |  |  |  |  |  |
| *Low* | — | — |  | — | — |  | — | — |  | — | — |  | — | — |  |
| *Medium* | 0.69 | 0.50, 0.95 | **0.025** | 0.65 | 0.46, 0.92 | **0.017** | 0.73 | 0.50, 1.05 | 0.086 | 0.76 | 0.53, 1.09 | 0.134 | 0.79 | 0.54, 1.16 | 0.219 |
| *High* | 0.54 | 0.39, 0.75 | **<0.001** | 0.53 | 0.37, 0.77 | **0.001** | 0.60 | 0.41, 0.88 | **0.010** | 0.62 | 0.42, 0.90 | **0.013** | 0.70 | 0.46, 1.07 | 0.096 |
| **TOTAL FEMUR** |  |  |  |  |  |  |  |  |  |  |  |  |  |  |  |
| *Low* | — | — |  | — | — |  | — | — |  | — | — |  | — | — |  |
| *Medium* | 0.71 | 0.51, 0.97 | **0.034** | 0.67 | 0.47, 0.94 | **0.022** | 0.77 | 0.54, 1.09 | 0.140 | 0.80 | 0.56, 1.14 | 0.213 | 0.82 | 0.56, 1.20 | 0.292 |
| *High* | 0.54 | 0.38, 0.75 | **<0.001** | 0.53 | 0.36, 0.77 | **0.001** | 0.62 | 0.43, 0.90 | **0.012** | 0.63 | 0.43, 0.91 | **0.016** | 0.69 | 0.45, 1.05 | 0.081 |
| ^1^Models: Model 1 was an unadjusted model; Model 2 was adjusted for age and race; Model 3 was further adjusted for education, poverty, BMI, sedentary behavior, history of smoking, and history of alcohol use based on Model 2; Model 4 included Model 3 and was further adjusted for family history of osteoporosis, and history of cortisone use, diabetes mellitus, and hypertension, and for estrogen use in women; Model 5 added dietary variables, including intake of VD, VC, Ca, P, Na, energy, protein, and caffeine, to model 4. Levels: Low level: <65.95 mcg/d for women, <69.85 mcg/d for men; Medium level: ≥65.95 mcg/d and <129.40 mcg/d for women, ≥69.85 mcg/d and <128.75 mcg/d for men; High level: ≥129.40 mcg/d for women, ≥128.75 mcg/d for men. | | | | | | | | | | | | | | | |
| ^2^*OR* = Odds Ratio, *CI* = Confidence Interval. | | | | | | | | | | | | | | | |

**Supplementary Table D Association between vitamin K intake levels and bone loss at different sites**

|  | Model 1 | | | Model 2 | | | Model 3 | | | Model 4 | | | Model 5 | | |
| --- | --- | --- | --- | --- | --- | --- | --- | --- | --- | --- | --- | --- | --- | --- | --- |
| **Characteristic**^1^ | ***OR***^2^ | **95% *CI***^2^ | ***P*** | ***OR***^2^ | **95% *CI***^2^ | ***P*** | ***OR***^2^ | **95% *CI***^2^ | ***P*** | ***OR***^2^ | **95% *CI***^2^ | ***P*** | ***OR***^2^ | **95% *CI***^2^ | ***P*** |
| **ALL PARTICIPANTS** | | | | | | | | | | | | | | | |
| **LUMBAR SPINE** |  |  |  |  |  |  |  |  |  |  |  |  |  |  |  |
| *Low* | — | — |  | — | — |  | — | — |  | — | — |  | — | — |  |
| *Medium* | 0.72 | 0.60, 0.88 | **0.001** | 0.72 | 0.59, 0.88 | **0.002** | 0.74 | 0.58, 0.93 | **0.012** | 0.74 | 0.59, 0.93 | **0.011** | 0.79 | 0.63, 0.99 | **0.041** |
| *High* | 0.77 | 0.65, 0.91 | **0.003** | 0.78 | 0.65, 0.94 | **0.009** | 0.79 | 0.65, 0.96 | **0.021** | 0.80 | 0.65, 0.97 | **0.024** | 0.86 | 0.70, 1.07 | 0.167 |
| **FEROMAL NECK** |  |  |  |  |  |  |  |  |  |  |  |  |  |  |  |
| *Low* | — | — |  | — | — |  | — | — |  | — | — |  | — | — |  |
| *Medium* | 0.78 | 0.62, 0.98 | **0.036** | 0.74 | 0.59, 0.94 | **0.014** | 0.75 | 0.58, 0.98 | **0.033** | 0.77 | 0.60, 0.99 | **0.039** | 0.77 | 0.59, 1.00 | **0.046** |
| *High* | 0.91 | 0.71, 1.16 | 0.427 | 0.89 | 0.70, 1.14 | 0.347 | 0.88 | 0.67, 1.17 | 0.383 | 0.91 | 0.69, 1.19 | 0.477 | 0.90 | 0.66, 1.24 | 0.519 |
| **TROCHANTER** |  |  |  |  |  |  |  |  |  |  |  |  |  |  |  |
| *Low* | — | — |  | — | — |  | — | — |  | — | — |  | — | — |  |
| *Medium* | 0.79 | 0.66, 0.94 | **0.009** | 0.77 | 0.64, 0.91 | **0.004** | 0.83 | 0.67, 1.02 | 0.077 | 0.85 | 0.69, 1.04 | 0.115 | 0.86 | 0.69, 1.08 | 0.188 |
| *High* | 0.88 | 0.68, 1.15 | 0.354 | 0.89 | 0.69, 1.14 | 0.351 | 0.97 | 0.71, 1.31 | 0.830 | 1.00 | 0.75, 1.33 | 0.985 | 1.04 | 0.75, 1.44 | 0.811 |
| **INTERTROCHANTER** |  |  |  |  |  |  |  |  |  |  |  |  |  |  |  |
| *Low* | — | — |  | — | — |  | — | — |  | — | — |  | — | — |  |
| *Medium* | 0.77 | 0.64, 0.92 | **0.005** | 0.73 | 0.61, 0.88 | **0.001** | 0.77 | 0.63, 0.94 | **0.011** | 0.79 | 0.65, 0.96 | **0.018** | 0.83 | 0.66, 1.04 | 0.099 |
| *High* | 0.80 | 0.64, 1.01 | 0.063 | 0.79 | 0.64, 0.99 | **0.040** | 0.81 | 0.64, 1.03 | 0.089 | 0.84 | 0.66, 1.06 | 0.138 | 0.92 | 0.70, 1.21 | 0.543 |
| **TOTAL FEMUR** |  |  |  |  |  |  |  |  |  |  |  |  |  |  |  |
| *Low* | — | — |  | — | — |  | — | — |  | — | — |  | — | — |  |
| *Medium* | 0.81 | 0.66, 0.99 | **0.037** | 0.78 | 0.64, 0.96 | **0.017** | 0.82 | 0.66, 1.02 | 0.074 | 0.84 | 0.68, 1.04 | 0.110 | 0.89 | 0.71, 1.12 | 0.311 |
| *High* | 0.84 | 0.65, 1.07 | 0.155 | 0.83 | 0.66, 1.05 | 0.121 | 0.86 | 0.66, 1.11 | 0.242 | 0.89 | 0.69, 1.14 | 0.348 | 0.97 | 0.73, 1.30 | 0.849 |
| ***MEN*** | | | | | | | | | | | | | | | |
| **LUMBAR SPINE** |  |  |  |  |  |  |  |  |  |  |  |  |  |  |  |
| *Low* | — | — |  | — | — |  | — | — |  | — | — |  | — | — |  |
| *Medium* | 0.89 | 0.67, 1.19 | 0.441 | 0.91 | 0.68, 1.22 | 0.509 | 0.92 | 0.67, 1.28 | 0.628 | 0.92 | 0.66, 1.28 | 0.617 | 0.83 | 0.59, 1.16 | 0.266 |
| *High* | 0.81 | 0.61, 1.08 | 0.149 | 0.82 | 0.61, 1.10 | 0.183 | 0.84 | 0.60, 1.18 | 0.130 | 0.85 | 0.61, 1.18 | 0.321 | 0.68 | 0.47, 0.99 | **0.044** |
| **FEROMAL NECK** |  |  |  |  |  |  |  |  |  |  |  |  |  |  |  |
| *Low* | — | — |  | — | — |  | — | — |  | — | — |  | — | — |  |
| *Medium* | 0.84 | 0.63, 1.12 | 0.220 | 0.80 | 0.59, 1.08 | 0.136 | 0.75 | 0.54, 1.03 | 0.071 | 0.74 | 0.54, 1.02 | 0.065 | 0.66 | 0.48, 0.90 | **0.011** |
| *High* | 1.02 | 0.74, 1.40 | 0.908 | 1.00 | 0.72, 1.39 | 0.998 | 0.92 | 0.63, 1.35 | 0.680 | 0.92 | 0.64, 1.33 | 0.646 | 0.78 | 0.52, 1.15 | 0.197 |
| **TROCHANTER** |  |  |  |  |  |  |  |  |  |  |  |  |  |  |  |
| *Low* | — | — |  | — | — |  | — | — |  | — | — |  | — | — |  |
| *Medium* | 1.07 | 0.79, 1.44 | 0.675 | 1.06 | 0.78, 1.43 | 0.709 | 1.19 | 0.84, 1.70 | 0.322 | 1.19 | 0.84, 1.70 | 0.327 | 1.09 | 0.76, 1.57 | 0.637 |
| *High* | 1.12 | 0.73, 1.74 | 0.594 | 1.13 | 0.74, 1.73 | 0.574 | 1.29 | 0.80, 2.06 | 0.288 | 1.30 | 0.83, 2.03 | 0.250 | 1.16 | 0.71, 1.90 | 0.545 |
| **INTERTROCHANTER** |  |  |  |  |  |  |  |  |  |  |  |  |  |  |  |
| *Low* | — | — |  | — | — |  | — | — |  | — | — |  | — | — |  |
| *Medium* | 1.03 | 0.74, 1.44 | 0.845  . | 1.02 | 0.73, 1.42 | 0.918  . | 1.09 | 0.72, 1.64 | 0.683  . | 1.08 | 0.71, 1.66 | 0.712  . | 1.05 | 0.65, 1.67 | 0.850  . |
| *High* | 1.09 | 0.74, 1.62 | 0.647 | 1.09 | 0.74, 1.60 | 0.668 | 1.15 | 0.72, 1.84 | 0.550 | 1.19 | 0.75, 1.88 | 0.447 | 1.13 | 0.65, 1.98 | 0.648 |
| **TOTAL FEMUR** |  |  |  |  |  |  |  |  |  |  |  |  |  |  |  |
| *Low* | — | — |  | — | — |  | — | — |  | — | — |  | — | — |  |
| *Medium* | 1.10 | 0.76, 1.57 | 0.613 | 1.09 | 0.76, 1.57 | 0.632 | 1.19 | 0.79, 1.77 | 0.396 | 1.19 | 0.80, 1.78 | 0.383 | 1.17 | 0.76, 1.78 | 0.466 |
| *High* | 1.08 | 0.73, 1.59 | 0.707 | 1.08 | 0.73, 1.58 | 0.703 | 1.15 | 0.72, 1.82 | 0.550 | 1.17 | 0.75, 1.83 | 0.472 | 1.13 | 0.68, 1.88 | 0.624 |
| ***WOMEN*** | | | | | | | | | | | | | | | |
| **LUMBAR SPINE** |  |  |  |  |  |  |  |  |  |  |  |  |  |  |  |
| *Low* | — | — |  | — | — |  | — | — |  | — | — |  | — | — |  |
| *Medium* | 0.59 | 0.45, 0.79 | **<0.001** | 0.57 | 0.42, 0.77 | **<0.001** | 0.56 | 0.40, 0.77 | **<0.001** | 0.57 | 0.41, 0.80 | **0.001** | 0.60 | 0.43, 0.83 | **0.003** |
| *High* | 0.70 | 0.54, 0.92 | **0.011** | 0.71 | 0.54, 0.94 | **0.016** | 0.68 | 0.50, 0.92 | **0.015** | 0.70 | 0.51, 0.96 | **0.030** | 0.78 | 0.57, 1.06 | 0.112 |
| **FEROMAL NECK** |  |  |  |  |  |  |  |  |  |  |  |  |  |  |  |
| *Low* | — | — |  | — | — |  | — | — |  | — | — |  | — | — |  |
| *Medium* | 0.73 | 0.54, 1.00 | **0.047** | 0.68 | 0.49, 0.95 | **0.023** | 0.70 | 0.49, 1.01 | 0.055 | 0.72 | 0.51, 1.03 | 0.068 | 0.74 | 0.52, 1.05 | 0.092 |
| *High* | 0.81 | 0.57, 1.16 | 0.247 | 0.80 | 0.57, 1.13 | 0.207 | 0.80 | 0.52, 1.23 | 0.309 | 0.85 | 0.55, 1.30 | 0.435 | 0.92 | 0.58, 1.47 | 0.725 |
| **TROCHANTER** |  |  |  |  |  |  |  |  |  |  |  |  |  |  |  |
| *Low* | — | — |  | — | — |  | — | — |  | — | — |  | — | — |  |
| *Medium* | 0.64 | 0.49, 0.84 | **0.001** | 0.61 | 0.46, 0.80 | **<0.001** | 0.62 | 0.46, 0.84 | **0.003** | 0.65 | 0.48, 0.88 | **0.007** | 0.66 | 0.48, 0.91 | **0.014** |
| *High* | 0.75 | 0.56, 1.01 | 0.056 | 0.76 | 0.57, 1.01 | 0.061 | 0.77 | 0.54, 1.11 | 0.159 | 0.82 | 0.57, 1.18 | 0.271 | 0.87 | 0.58, 1.30 | 0.499 |
| **INTERTROCHANTER** |  |  |  |  |  |  |  |  |  |  |  |  |  |  |  |
| *Low* | — | — |  | — | — |  | — | — |  | — | — |  | — | — |  |
| *Medium* | 0.64 | 0.50, 0.81 | **<0.001** | 0.59 | 0.46, 0.76 | **<0.001** | 0.60 | 0.47, 0.78 | **<0.001** | 0.63 | 0.49, 0.81 | **<0.001** | 0.66 | 0.50, 0.86 | **0.003** |
| *High* | 0.67 | 0.52, 0.85 | **0.002** | 0.65 | 0.50, 0.84 | **0.001** | 0.63 | 0.48, 0.83 | **0.001** | 0.66 | 0.51, 0.86 | **0.003** | 0.72 | 0.54, 0.96 | **0.027** |
| **TOTAL FEMUR** |  |  |  |  |  |  |  |  |  |  |  |  |  |  |  |
| *Low* | — | — |  | — | — |  | — | — |  | — | — |  | — | — |  |
| *Medium* | 0.65 | 0.51, 0.84 | **0.001** | 0.61 | 0.47, 0.80 | **<0.001** | 0.61 | 0.46, 0.82 | **0.001** | 0.65 | 0.49, 0.86 | **0.003** | 0.68 | 0.51, 0.91 | **0.010** |
| *High* | 0.70 | 0.53, 0.93 | **0.015** | 0.69 | 0.52, 0.92 | **0.012** | 0.67 | 0.48, 0.92 | **0.015** | 0.71 | 0.52, 0.98 | **0.040** | 0.80 | 0.57, 1.13 | 0.195 |
| ^1^Models: Model 1 was an unadjusted model; Model 2 was adjusted for age and race; Model 3 was further adjusted for education, poverty, BMI, sedentary behavior, history of smoking, and history of alcohol use based on Model 2; Model 4 included Model 3 and was further adjusted for family history of osteoporosis, and history of cortisone use, diabetes mellitus, and hypertension, and for estrogen use in women; Model 5 added dietary variables, including intake of VD, VC, Ca, P, Na, energy, protein, and caffeine, to model 4. Levels: Low level: <65.95 mcg/d for women, <69.85 mcg/d for men; Medium level: ≥65.95 mcg/d and <129.40 mcg/d for women, ≥69.85 mcg/d and <128.75 mcg/d for men; High level: ≥129.40 mcg/d for women, ≥128.75 mcg/d for men. | | | | | | | | | | | | | | | |
| ^2^*OR* = Odds Ratio, *CI* = Confidence Interval. | | | | | | | | | | | | | | | |

**
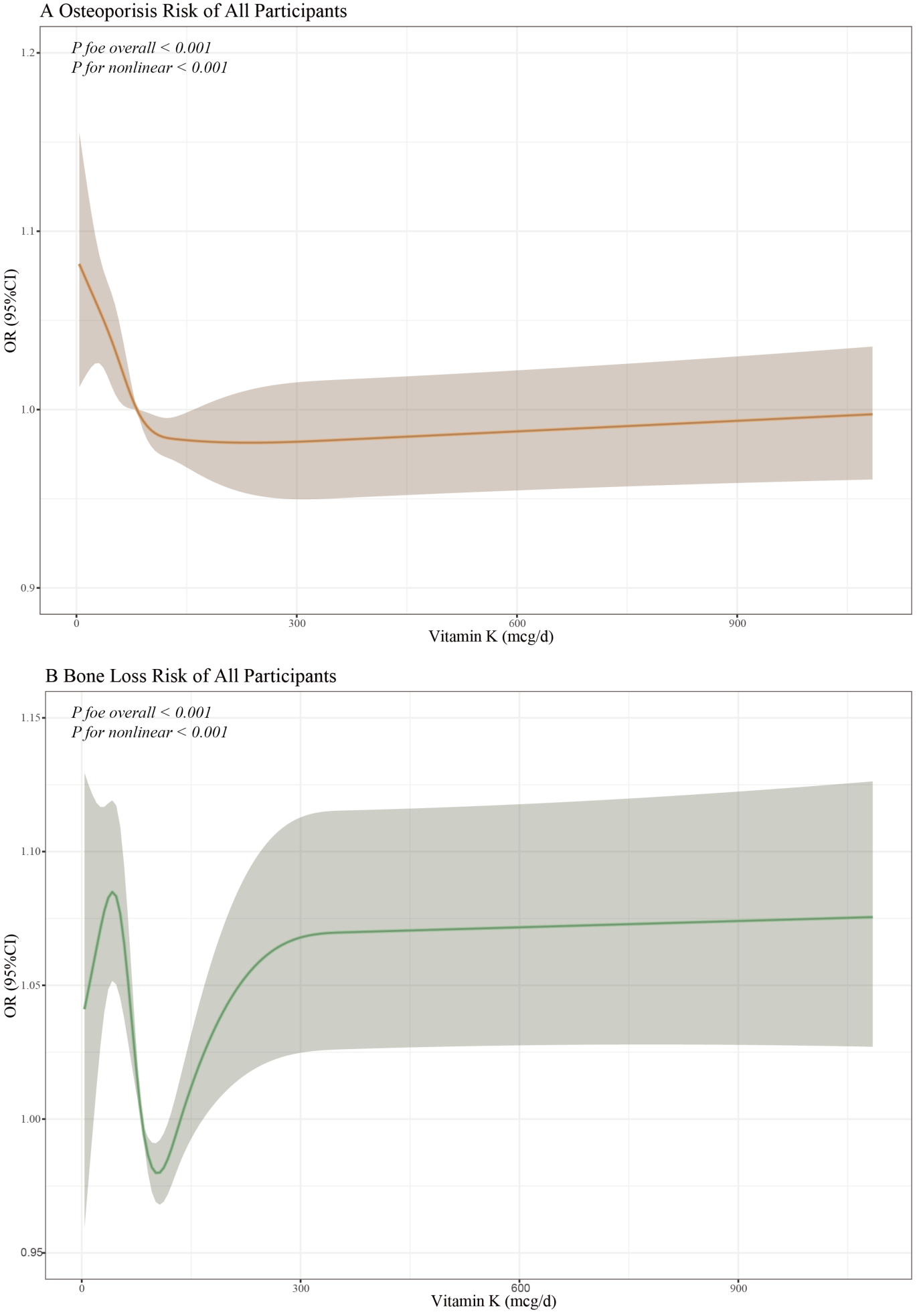
Supplementary Figure 1 The association between vitamin K intake and bone health.**

*OR* = Odds Ratio, *CI* = Confidence Interval.
